# Assessment of Mpox knowledge and attitudes among health workers in Egypt and Arab countries based on a national survey and a meta-analysis

**DOI:** 10.1038/s41598-025-28446-z

**Published:** 2025-12-04

**Authors:** Neamat Hamdy Elsawy, Rasha Ashmawy, Alaa Abd Elkader Abd Elhady Elmallah, Moshira Mansour Abd Ellatif, Sarah Magdy Amin Mokhtar, Ramzeya Mohamed Abdelwahab, Samar Saeed Abdo Elsronbaw, Aisha Elsharkawy, Ehab Kamal, Marwa Rashad Salem

**Affiliations:** 1 Clinical Pharmacy Inspection, Fowa Health Administration, Ministry of Health and Population, Kafr Elsheikh, Egypt; 2 PhD student at Medical Research Institute, Alexandria University, Alexandria, Egypt; 3 Department of Clinical Research, Maamora Chest Hospital, Ministry of Health and Population, Alexandria, Egypt; 4 Scientific Committees’ Department at the Egyptian Health Council, Cairo, Egypt; 5 Head of Pharmacy Department, Akhmim Health Administration, Ministry of Health and Population, Sohag, Egypt; 6 PICU Clinical Pharmacy, Mansheyet El Bakry General Hospital, Ministry of Health and Population, Cairo, Egypt; 7 Department of Clinical Research, Abou-kir General Hospital, Ministry of Health and Population, Alexandria, Egypt; 8 Department of Obstetrics and Gynecology, Fuwa, Health Insurance Organization, Survey Collection&Methodology, Kafr ElSheikh, Egypt; 9 Endemic Medicine and Hepatology Department, Faculty of Medicine, Cairo University, Cairo, Egypt; 10 Medical Research Division, National Research Center (NRC), Giza, Egypt; 11 Department of Public Health and Community Medicine, Cairo University, Cairo, Egypt

**Keywords:** Mpox; monkeypox, Health care workers, Knowledge, Attitude, Egypt, Arab countries, Meta-analysis, Public health preparedness, Diseases, Health care, Medical research, Risk factors

## Abstract

Recent Mpox outbreaks in non-endemic countries have highlighted the importance of global health preparedness. Combined national survey and pooled analysis were conducted to assess the knowledge and attitudes of healthcare workers (HCWs) toward Mpox in Egypt and the Arab region. An online survey was distributed to HCWs in Egypt, and a literature search of PubMed and other sources was performed to identify relevant studies from Arab countries. Descriptive statistics were utilized for all variables, with chi-square and t-tests used for comparisons. A random-effects meta-analysis was performed, with heterogeneity assessed using the I^2^ statistic. Subgroup analyses were conducted to explore factors of heterogeneity. A total of 399 eligible HCWs from various Egyptian health facilities were included. The mean age of participants was 35.6 ± 7.1 years; the majority were female, married, pharmacists, and had at least five years of experience. The survey revealed that only 37.6% of Egyptian HCWs had good knowledge of Mpox, while 97.9% held positive attitudes. The meta-analysis of 30 studies from Arab countries including both HCWs and the general population, showed a pooled proportion of 35% (95% CI: 31%–39%) for good knowledge and 48% (95% CI: 37%–59%) for positive attitude. A higher positive attitude was significantly associated with female gender and HCWs. Significant knowledge gaps regarding Mpox exist among HCWs in Egypt and the wider Arab region, despite generally positive attitudes. This underscores an urgent need to update medical curricula, implement continuous educational programs and launch nationwide awareness campaigns.

**Supplementary Information:**

The online version contains supplementary material available at 10.1038/s41598-025-28446-z.

## Introduction

Monkeypox (Mpox) is a zoonotic infectious disease caused by the Mpox virus, a double-stranded DNA virus of the Orthopoxvirus genus and family^1^. First identified in 1958, human infection was initially reported in the Democratic Republic of the Congo in 1970^2^. The current global outbreak of human Mpox virus infection is characterized by changes in the viral biological nature, the behavior of individuals, or maybe both^3^. The clinical presentation of the disease resembles smallpox but with less severity. Transmission to humans can occur through direct contact with infected animals or people, contaminated materials, respiratory droplets, consumption of infected meat, or sexual contact, particularly among men who have sex with men (MSM)^4^.

From January 2022 to April 2025, countries outside the African region accounted for 71.5% of all Mpox cases. Moreover, since 1 January 2022, cases of Mpox have been reported to WHO from 30 African member states. As of 6 July 2025, a total of 46 589 laboratory-confirmed cases, including 189 deaths, have been reported to WHO^5^. Egypt reported its first confirmed case of Mpox in September 2022, involving a man returning from Spain. According to the World Health Organization (WHO), the total number of confirmed cases in Egypt remains low, with only three cases reported to date^5^.

Adequate knowledge and positive attitudes among healthcare workers (HCWs) are essential for effective disease management, as outlined in the Knowledge, Attitudes, and Practices (KAP) framework^6^. This is critically important for Mpox as WHO report identified inadequate knowledge as a barrier preventing its reemergence^7^. With the recent increase of Mpox cases, enhancing the ability of HCWs to detect cases and manage patients is a crucial feature of the surveillance system^8^. However, studies from Arab countries consistently report significant gaps. HCWs in Saudi Arabia, Lebanon, and Kuwait demonstrate inadequate Mpox knowledge and suboptimal attitudes^7,9,10^ Furthermore, a multicenter cross-sectional study reported poor knowledge level and attitude among nursing students, with 57.2 % reporting moderate anxiety^11^. Similarly, an Egyptian study found that only 55.3% of HCWs and medical students had adequate knowledge, while less than half exhibited favorable attitudes^12^. Another reported just 7.3% with good knowledge^8^. Besides the strategic geographic location of Egypt in North Africa, the country serves as both a major travel hub and a host nation for international students and refugees^13^.

Although individual studies highlight gaps in Mpox knowledge and attitudes, the existing literature remains fragmented, lacking a comprehensive regional synthesis. No study has yet provided a unified assessment to quantify the magnitude of this awareness gap across Arab countries or applied a theoretical framework to identify common determinants of poor knowledge and attitudes^14,15^. To bridge this gap, the present study adopts a dual-method approach guided by the KAP framework to evaluate Mpox-related knowledge and attitudes among Egyptian healthcare workers. The findings will also contribute to a regional pooled analysis to estimate overall awareness levels and identify shared risk factors. This evidence aims to guide targeted educational interventions and support policymakers in enhancing regional preparedness against Mpox threats.

## Methods

### The study was conducted in two phases

#### Phase one

The current study is an exploratory e-open survey that was performed among the health care providers to assess their M pox related knowledge and attitudes. The research was carried out in accordance with the Checklist for Reporting Results of Internet E Surveys (CHERRIES) guidelines^16^.

#### Phase two

A meta-analysis was conducted by pooling the results of the current study with findings from similar studies conducted in other Arabic countries. This phase aimed to estimate the overall level of Mpox-related knowledge and attitudes in the region, identify common knowledge gaps, and determine potential risk factors associated with low awareness. The combined analysis provides a broader regional perspective to support evidence-based interventions and inform public health decision-making.

### Phase one

#### Study population and setting

The study targeted Egyptian healthcare providers, including physicians, pharmacists, high-level nursing staff, and other medical professionals. Participants were eligible for inclusion if they met the following criteria: (i) being an Egyptian physician, pharmacist, or high-level nursing staff member currently employed by the MoHP; (ii) being 18 years of age or older; and (iii) voluntarily agreeing to participate by providing electronically signed informed consent.

#### Data collection tool and procedure

Data were collected using a Google Form questionnaire adapted from a previous cross-sectional study conducted in Saudi Arabia on physicians’ knowledge and attitudes toward Mpox. The survey link was distributed electronically through various social media platforms (WhatsApp, Telegram, Facebook, Instagram) and via email, targeting healthcare providers across all Egyptian governorates. A convenience sampling method was employed to recruit participants from September 2024 till March 2025.

#### Sample size

The required sample size was calculated using Stata Statistical Software version 16. Based on the most recent data from the Central Agency for Public Mobilization and Statistics (CAPMAS), the estimated number of healthcare workers in Egypt in 2021 was approximately 375,000^17^. Referring to a previous study conducted in Saudi Arabia, where physician knowledge of Mpox was estimated at 55%, this figure was used as the expected prevalence^3^.To achieve a 95% confidence level and a 5% margin of error, the minimum required sample size was calculated to be 380 participants.

#### Data collection tool

The data were collected using a structured questionnaire adapted from a previously published study that assessed healthcare professionals’ knowledge and attitudes toward the Mpox virus in Saudia Arabia^18^. The instrument was slightly modified to align with the Egyptian healthcare context while maintaining the conceptual integrity of the original version.

The questionnaire consisted of four main sections:

1. Sociodemographic information:
  Included questions on age, gender, marital status, professional role, medical specialty, sector of employment, and years of medical experience.
2. Professional Background:
  Collected additional information regarding the participants’ current medical practice and exposure to infectious disease-related training.
3. Knowledge Assessment:
  Comprised multiple-choice questions (MCQs) regarding Mpox. Correct answers received a score of 1, while incorrect answers scored 0. The total knowledge score ranged from 0 to 22, with higher scores indicating greater knowledge.
4. Attitude Assessment:
  Consisted of ten statements evaluated using a 3-point Likert scale (agree, neutral, disagree). Statements addressed participants’ views on the global capacity to control Mpox, adequacy of preventive measures, emotional reactions to the outbreak, and interest in learning more about Mpox, emerging diseases, and travel medicine.

A pilot study was conducted on 20 participants to evaluate the clarity, cultural relevance, and comprehensibility of the adapted questionnaire. Feedback from the pilot participants was used to refine the survey items and ensure content validity within the Egyptian healthcare setting.

The internal consistency of the finalized instrument was assessed using Cronbach’s alpha (α), yielding a value of **0.79**, indicating good reliability and confirming that the questionnaire was suitable for use in the main study.

### Phase two

#### Search strategy

Two researchers conducted a literature search in PubMed, supplemented by manual searches. Keywords such as "Monkeypox," "knowledge," and “attitude” were used in the search strategy. The initial search started on March 15, 2025, and was updated until May 3, 2025.

Eligible studies for the meta-analysis included all observational studies conducted between 2022 and 2025 in Arab countries that assessed the prevalence of knowledge and/or attitudes toward Mpox among healthcare professionals, medical students, or the general population. Studies were excluded if they were systematic reviews, conference abstracts, case reports, or case series; if they did not address the primary objectives related to Mpox knowledge or attitudes; or if they lacked sufficient data or did not report the necessary outcomes for inclusion in the pooled analysis.

#### Data extraction

Two independent researchers meticulously collected relevant data from the selected articles. The following details were extracted and recorded in an Excel spreadsheet: first author’s name, publication year, country, sample size, study population, gender (male and female), mean age, study period, the number of Mpox good knowledge, and the number of positive attitudes towards Mpox. Finally, a third researcher verified the extracted data to ensure accuracy and eliminate incorrect information.

### Statistical analysis

Descriptive statistics including percentages and frequency distributions were calculated. Chi-square tests were primarily used to compare proportional differences in all responses between gender groups, with Fisher’s exact tests applied when cell counts were small. For continuous variables, Student’s t-test or Mann-Whitney U tests were utilized to assess bivariate analysis according to data normality. The significance threshold was set at p<0.05 for all analyses.

The meta-analysis employed random-effects models using the DerSimonian-Laird estimator to calculate pooled proportions and 95% confidence intervals for both knowledge and attitude outcomes, while quantifying heterogeneity through I^2^ statistics and tau^2^ estimates. Subgroup analyses were conducted using mixed-effects meta-regression models to examine potential moderators (year of data collection, country, population type, gender distribution, and mean age), with statistical significance set at p<0.05, all implemented in R version 4.2.2 using the meta and metafor packages. The random-effects approach was chosen a priori due to expected clinical and methodological heterogeneity across studies, with all statistical tests being two-tailed.

## Results

### HCWs survey

The study surveyed 399 healthcare workers, predominantly female (82.0%), the average age was 35.6 ± 7.1 years, with most respondents falling into the 21–35 age group 217 (54.4%), followed by 36–50 years 171 (42.9%), and a small proportion over 50, the majority were married 289 (72.4%).

Professionally, pharmacists constituted the largest group 274 (68.7%), followed by physicians (23.8%), nurses (5.8%), and dentists (1.8%). Nearly half (45.6%) were specialists, while 36.6% were general practitioners, 13.3% consultants, and 4.5% residents. A significant proportion (73.4%) had over five years of experience. Geographically, most participants were from the Delta region (57.9%), followed by Alexandria (19.0%), Greater Cairo (17.5%), and smaller numbers from the Canal region (3.3%) and Upper Egypt (2.3%).

Regarding Mpox awareness, 83.2% had heard of the disease, though only 46.1% received formal medical education on it. Most (78.9%) learned about M. pox a month or more prior, Table 1 and Figure S1.

**Table 1:** Sociodemographic characteristics of respondents

Total (N=399)	N	%
Age, Years, Mean ± SD	35.6 ± 7.1	
Age category		
21 – 35	217	54.4
36 – 50	171	42.9
>50	11	2.8
Marital status		
Married	289	72.4
Single	89	22.3
Divorced/widow	21	5.3
Position		
Pharmacist	274	68.7
Physician	95	23.8
Nurse	23	5.8
Dentist	7	1.8
Level of work		
Specialist	182	45.6
General Practitioner	146	36.6
Consultant	53	13.3
Residents	18	4.5
Medical experience		
Less than one year	27	6.8
1-5 years	79	19.8
More than five years	293	73.4
Region		
Delta region	231	57.9
Alexandria region	76	19
Great Cairo region	70	17.5
Canal Region	13	3.3
Upper Egypt	9	2.3
Mpox medical education, (yes)	184	46.1
Heard about Mpox before, (yes)	332	83.2
First time knowing Mpox		
I didn’t hear about it	27	6.8
Within several days or weeks ago	57	14.3
Within the last month or later	315	78.9

**Fig. 1:**
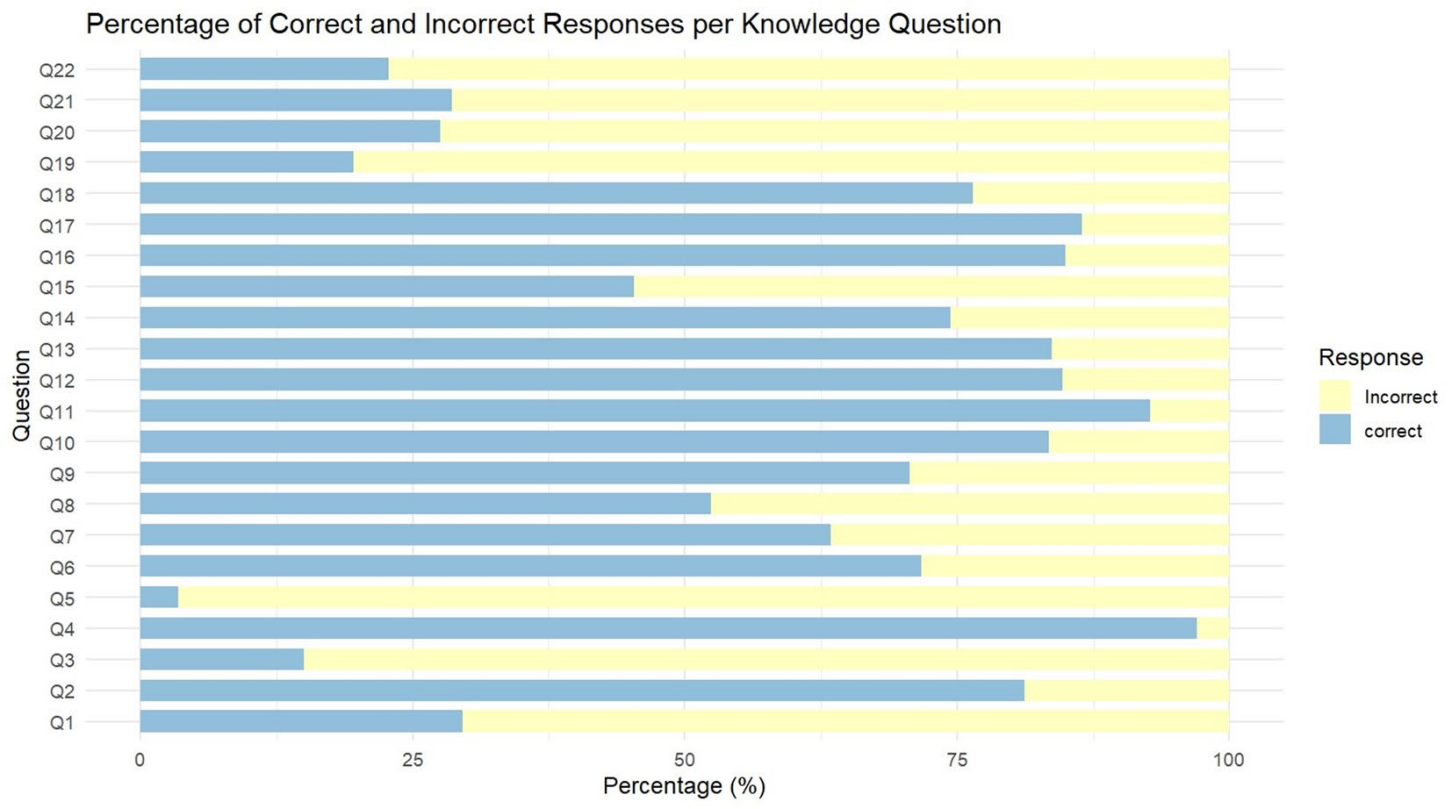

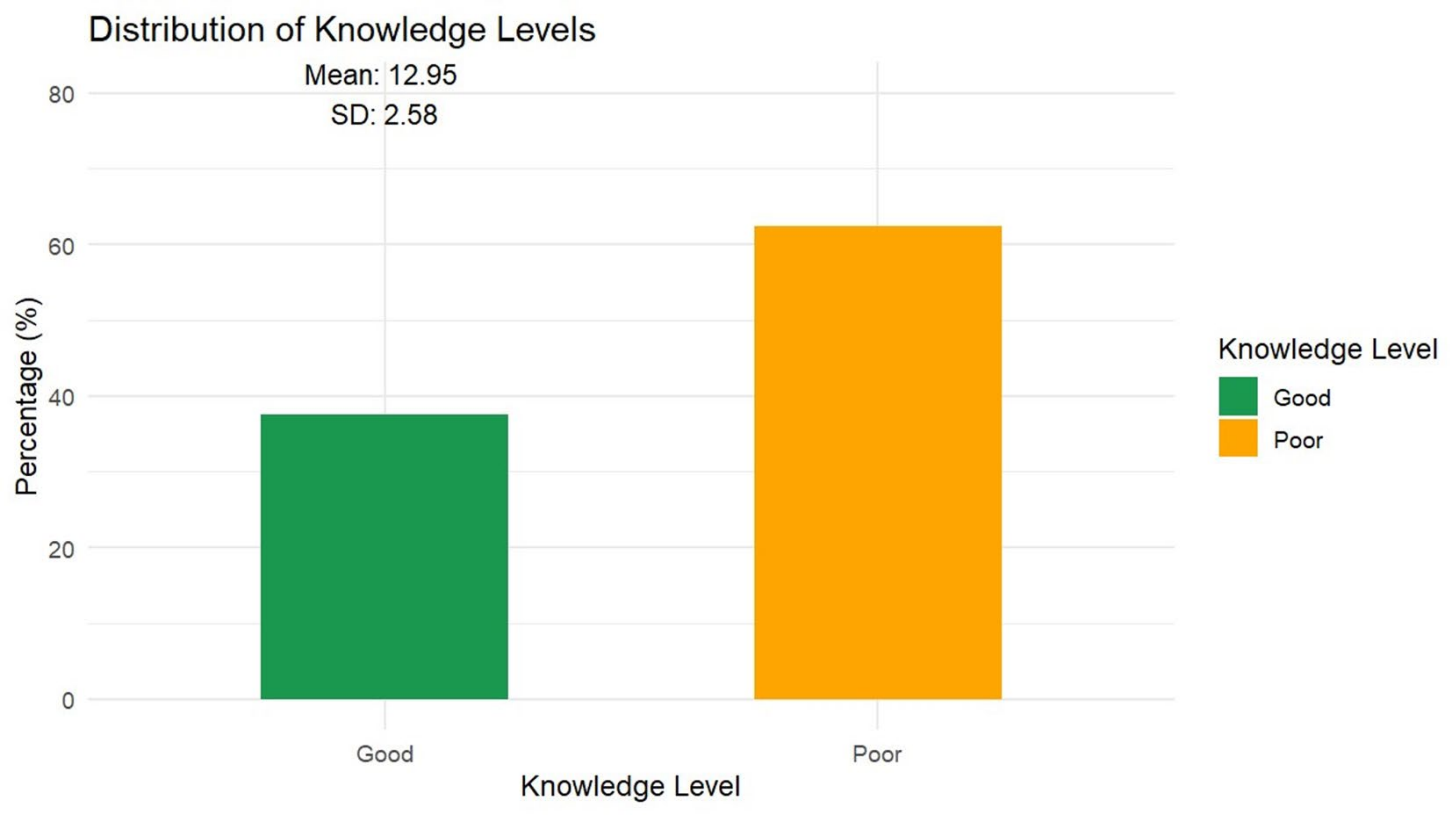
(**A**) Knowledge questions percentage of correct answer distribution. (**B**) distribution of good vs. poor knowledge among respondents.

Figures 1A, 1B and Table S2 evaluate healthcare workers’ knowledge of monkeypox across 22 questions, comparing overall and gender-based responses. Overall knowledge was moderate, with a mean score of 13.0 ± 2.6 (median: 13.0) out of a possible 22, and only 37.6% demonstrating “good” knowledge. No significant gender differences existed in total knowledge scores (p = 0.774).

For basic virology and transmission, nearly all respondents correctly identified monkeypox as a viral disease (97.0%), with no gender differences (p = 0.900). However, 70.4% incorrectly believed monkeypox is prevalent in Middle Eastern countries, and 85.0% wrongly assumed high case numbers in Egypt. Most knew it is endemic in Africa (81.2%), but only 62.5% of males (vs. 73.7% females, p = 0.056) recognized its human-to-human transmissibility.

Regarding clinical features and diagnosis, high accuracy was observed for rash-related symptoms (92.7%) and lymphadenopathy as a key differentiating sign (85.0%). Flu-like symptoms (83.5%) and skin lesions (papules/vesicles/pustules, 74.4–84.7%) were well-recognized. Diarrhea as a symptom was underrecognized (45.4% correct), with males scoring higher (55.6% vs. 43.1% females, p = 0.055).

Then for knowledge of Mpox management and vaccination 86.5% correctly endorsed paracetamol for symptomatic management, but 29.2% of males (vs. 17.4% females,

p = 0.023) erroneously believed antibiotics are required. Misinformation about vaccines was common: 72.4% incorrectly thought chickenpox vaccine protects against monkeypox, and 71.4% were unaware of a specific monkeypox vaccine.

Table 2 presents the distribution of Mpox knowledge levels among 399 healthcare workers, of whom 150 (37.6%) demonstrated *good* knowledge. No statistically significant differences in knowledge were observed across gender (p = 0.774), age categories (p = 0.994), marital status (p = 0.510), occupational position (p = 0.739) or region (p = 0.201). Nevertheless, a regional variation was observed, as participants from the Delta region exhibited the highest proportion of good knowledge (42.4%), whereas those from the Canal region recorded the lowest (23.1%). Although the association between professional degree and knowledge was not statistically significant (p = 0.066), notable trends were observed. General practitioners achieved the highest proportion of good knowledge (45.9%), followed by residents (38.9%), while consultants (30.2%) showed comparatively lower levels of good knowledge.

**Table 2:** Factors associated with Mpox knowledge levels among healthcare workers (N=399).

n (%)	Total (N=399)	Good (n=150)	Poor (n=249)	p-value
Sex				0.774
Female	327	124 (37.9%)	203 (62.1%)	
Male	72	26 (36.1%)	46 (63.9%)	
Age Category, years				0.994
21–35	217	82 (37.8%)	135 (62.2%)	
36–50	171	64 (37.4%)	107 (62.6%)	
>50	11	4 (36.4%)	7 (63.6%)	
Marital status				0.510
Married	289	105 (36.3%)	184 (63.7%)	
Single	89	38 (42.7%)	51 (57.3%)	
Divorced/widow	21	7 (33.3%)	14 (66.7%)	
Position				0.739
Pharmacist	274	102 (37.2%)	172 (62.8%)	
Physician	95	34 (35.8%)	61 (64.2%)	
Nurse	23	11 (47.8%)	12 (52.2%)	
Dentist	7	3 (42.9%)	4 (57.1%)	
Level of work				0.066
Specialist	182	60 (33.0%)	122 (67.0%)	
General Practitioner	146	67 (45.9%)	79 (54.1%)	
Consultant	53	16 (30.2%)	37 (69.8%)	
Residents	18	7 (38.9%)	11 (61.1%)	
Medical experience, years				0.005*
Less than one year	27	16 (59.3%)	11 (40.7%)	
1–5 years	79	37 (46.8%)	42 (53.2%)	
More than five years	293	97 (33.1%)	196 (66.9%)	
Region				0.201
Delta region	231	98 (42.4%)	133 (57.6%)	
Alexandria region	76	25 (32.9%)	51 (67.1%)	
Great Cairo region	70	21 (30.0%)	49 (70.0%)	
Canal Region	13	3 (23.1%)	10 (76.9%)	
Upper Egypt	9	3 (33.3%)	6 (66.7%)	
Mpox medical education				<0.001*
No	215	62 (28.8%)	153 (71.2%)	
Yes	184	88 (47.8%)	96 (52.2%)	
Heard about Mpox before				0.743
No	67	24 (35.8%)	43 (64.2%)	
Yes	332	126 (38.0%)	206 (62.0%)	
First time knowing Mpox				0.166
I didn’t hear about it	27	13 (48.1%)	14 (51.9%)	
Within several days or weeks ago	57	26 (45.6%)	31 (54.4%)	
aWithin the last month or later	315	111 (35.2%)	204 (64.8%)	

Knowledge was assessed using a mean score out of 13 and classified as good or poor.

The chi-square test or exact test was used to determine statistical significance.

A p-value<0.05 was considered statistically significant.

A notable inverse relationship emerged between years of experience and knowledge scores (p = 0.005); participants with less than one year of experience had the highest rate of good knowledge (59.3%), which declined to 46.8% among those with 1–5 years and 33.1% among those with more than five years of experience. Moreover, prior formal education or training on Mpox showed a highly significant association with knowledge levels (p < 0.001). Nearly half of trained participants (47.8%) demonstrated good knowledge, compared to less than one-third of untrained staff (28.8%). In contrast, prior awareness of Mpox did not significantly influence knowledge levels (p = 0.743).

**Fig. 2:**
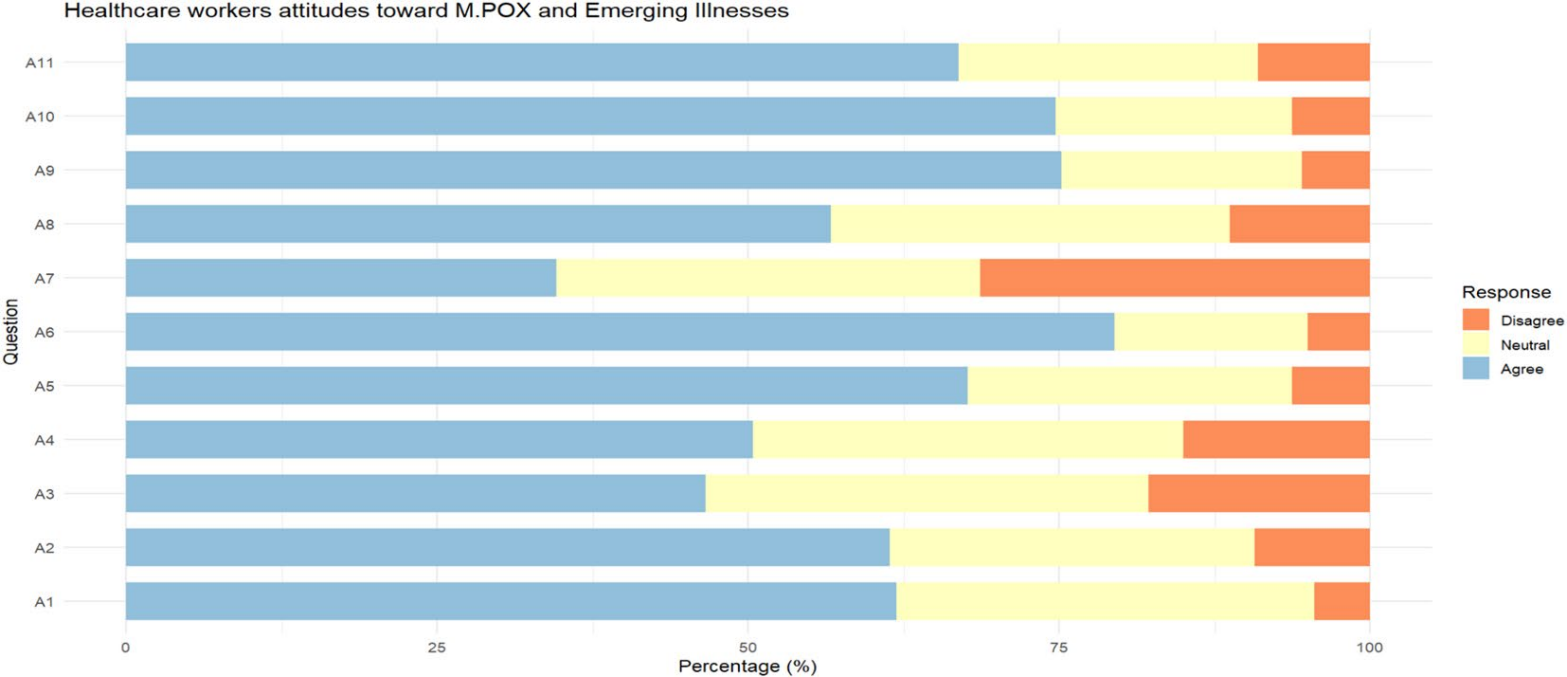

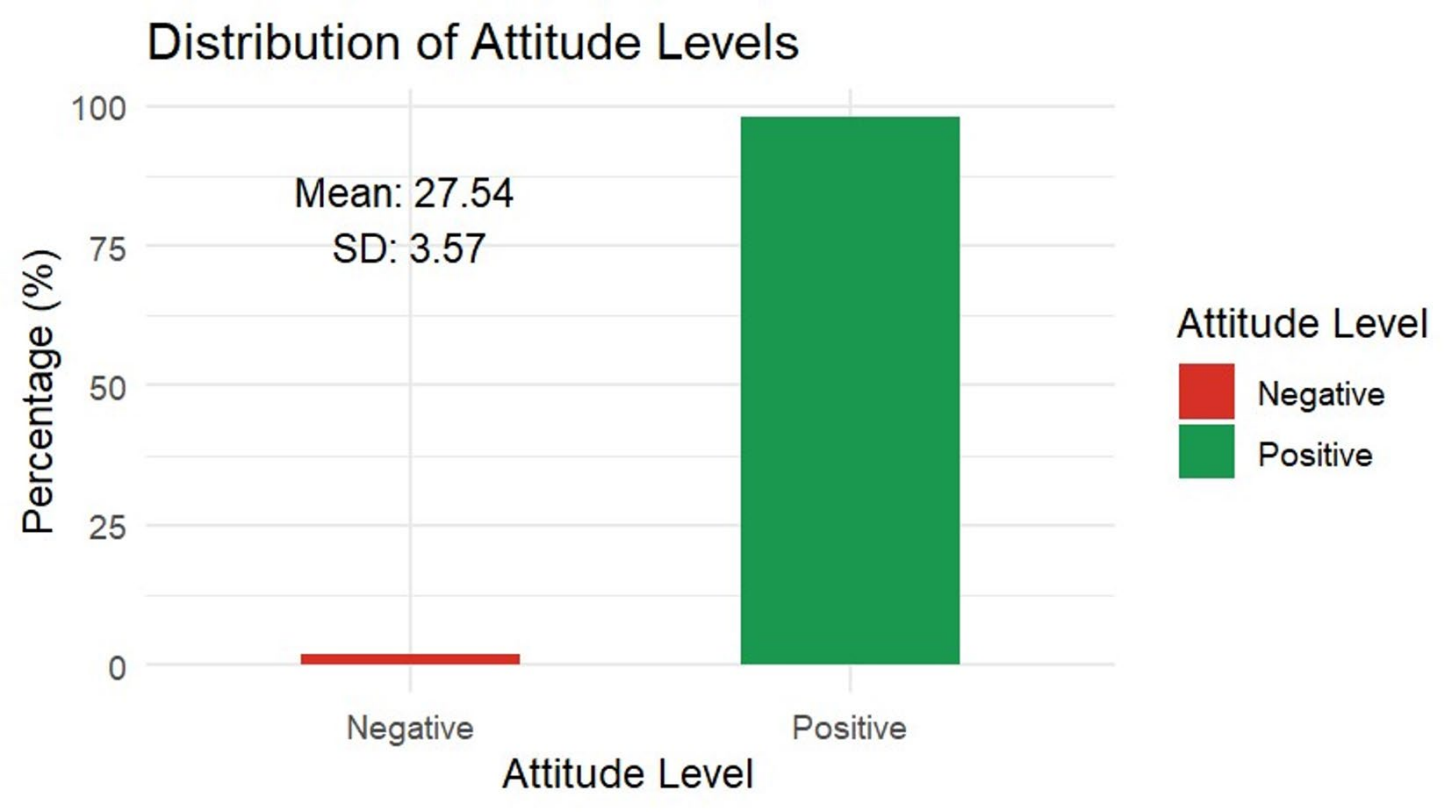
(**A**) Attitude questions percentage of correct answer distribution. (**B**) distribution of positive vs negative attitude among respondents.

Figure 2A–B and supplementary Table S3 illustrate healthcare workers’ attitudes toward Mpox management. Overall, the findings indicate a predominantly positive outlook, with 391 (97.9%) of participants expressing favorable attitudes. Most respondents demonstrated confidence in control measures, as 61.9% believed the global population could contain Mpox worldwide, and 61.4% expressed confidence in the Egyptian MoHP and local communities to control the disease domestically. Additionally, nearly half (46.6%) agreed that current prevention and control measures are sufficient.

Perceived risk varied among participants: 50.4% believed Mpox could spread to Egypt, and 34.6% were concerned it might evolve into a worldwide pandemic. Despite these concerns, a substantial proportion of participants expressed interest in continued learning, with 79.4% wanting more knowledge about Mpox, 75.2% seeking education on emerging diseases, and 74.7% interested in Travel Medicine training.

Significant gender differences were observed in specific attitude domains. Male participants were more likely to emphasize the role of mass media in global prevention (81.9% vs. 64.5%, p = 0.011), yet they were less concerned about the healthcare system burden (45.8% vs. 59.0%, p = 0.045) and travel-related risks (56.9% vs. 69.1%, p = 0.027) compared to females. Despite these differences, both genders shared a comparably strong enthusiasm for further education on Mpox and related topics.

### Meta analysis of pooled knowledge and attitude in Arab countries

A total of 135 records were initially identified through the PubMed search, and after applying language and human study filters, 65 studies remained. An additional five studies were retrieved through manual search. Following the screening and exclusion of 40 irrelevant or ineligible studies, 30 studies met the inclusion criteria and were included in the final analysis, Supplementary Tables S4. A detailed summary of the characteristics of the included studies is provided in Supplementary Table S5.

**Fig. 3:**
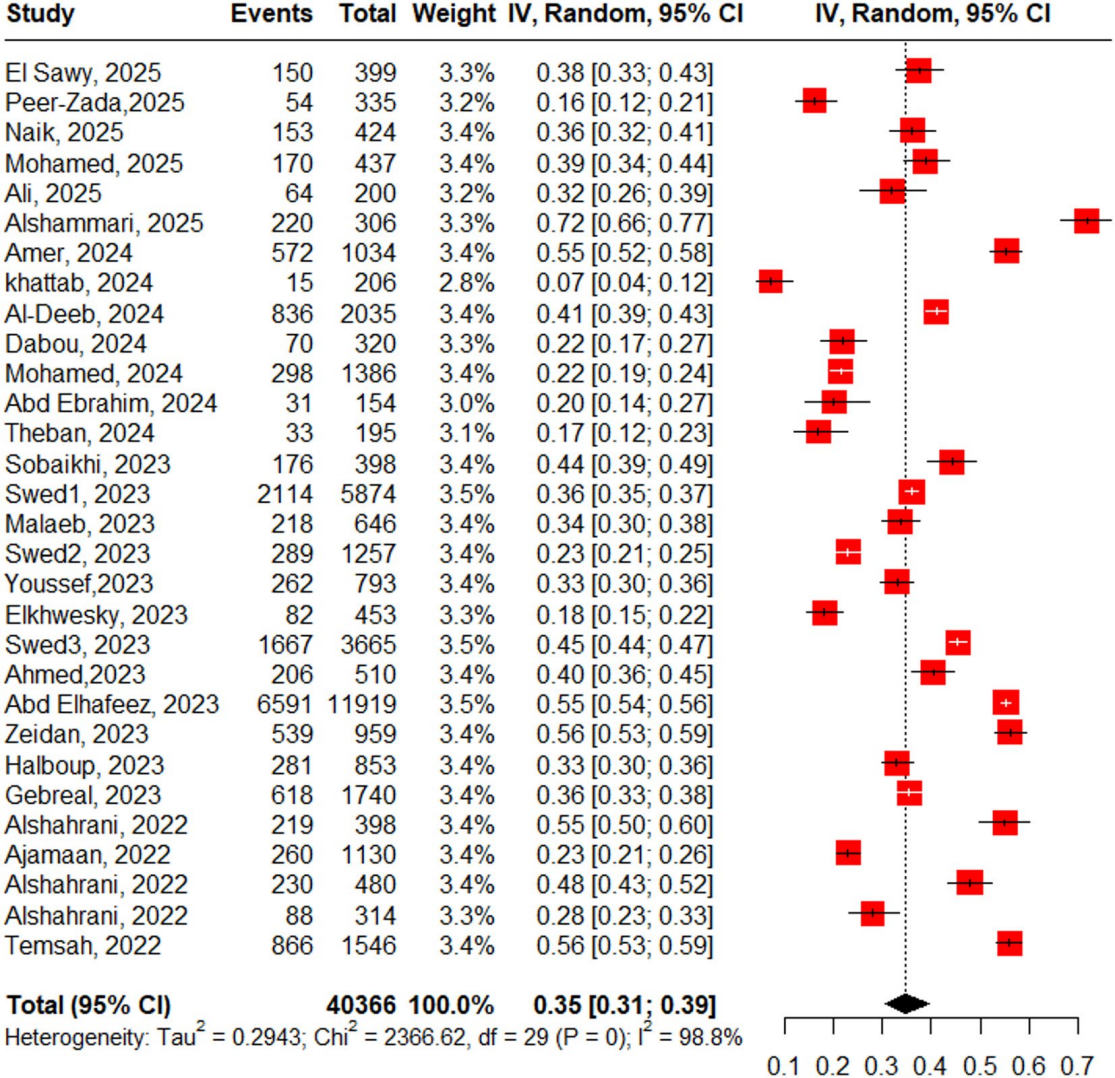
Forest plot of pooled M. Pox good knowledge percentage among Arabic population.

Figure 3 displays a forest plot summarizing a meta-analysis of 30 studies evaluating Mpox knowledge among 40366 populations in Arab countries. The pooled analysis reveals that only 35% (95% CI: 31%−39%) of participants demonstrated adequate knowledge, indicating generally poor awareness throughout the region. The analysis showed substantial heterogeneity (I^2^ = 98.8%), reflecting considerable variation in knowledge levels attributable to factors such as country-specific contexts, study populations (healthcare workers versus general public), timing of data collection, and gender distribution of samples. Reported knowledge levels ranged from low as 7% (Khattab, 2024) to high as 72% (Alshammari, 2025). The Egger’s test shows significant funnel plot asymmetry (z = −3.38, p = 0.0007), suggesting possible publication bias or small-study effects. While the adjusted effect size remains positive (b = 0.729).

Subgroup analyses identified the year of data collection as a significant moderator of knowledge levels (p=0.015), with studies from 2023 showing the lowest pooled knowledge estimate (25%, 95% CI: 18%−33%) and those from 2024 demonstrating marked improvement (46%, 95% CI: 33%−59%), Figure S 6.1. Regarding country, Saudi Arabia contributed the largest number of studies (n=12) and showed the highest pooled knowledge (38%, 95% CI: 30%−46%), Egypt’s five studies yielded the lowest estimate (25%, 95% CI: 16%−36%), though these country-level differences were not statistically significant (p=0.669), Figure S 6.2. Additional subgroup analyses found no significant associations between knowledge levels and gender distribution (p=0.477), population type (p=0.393), or mean participant age (p=0.972), Supplementary Table S6.3-Supplementary Table S6.5.

**Fig. 4:**
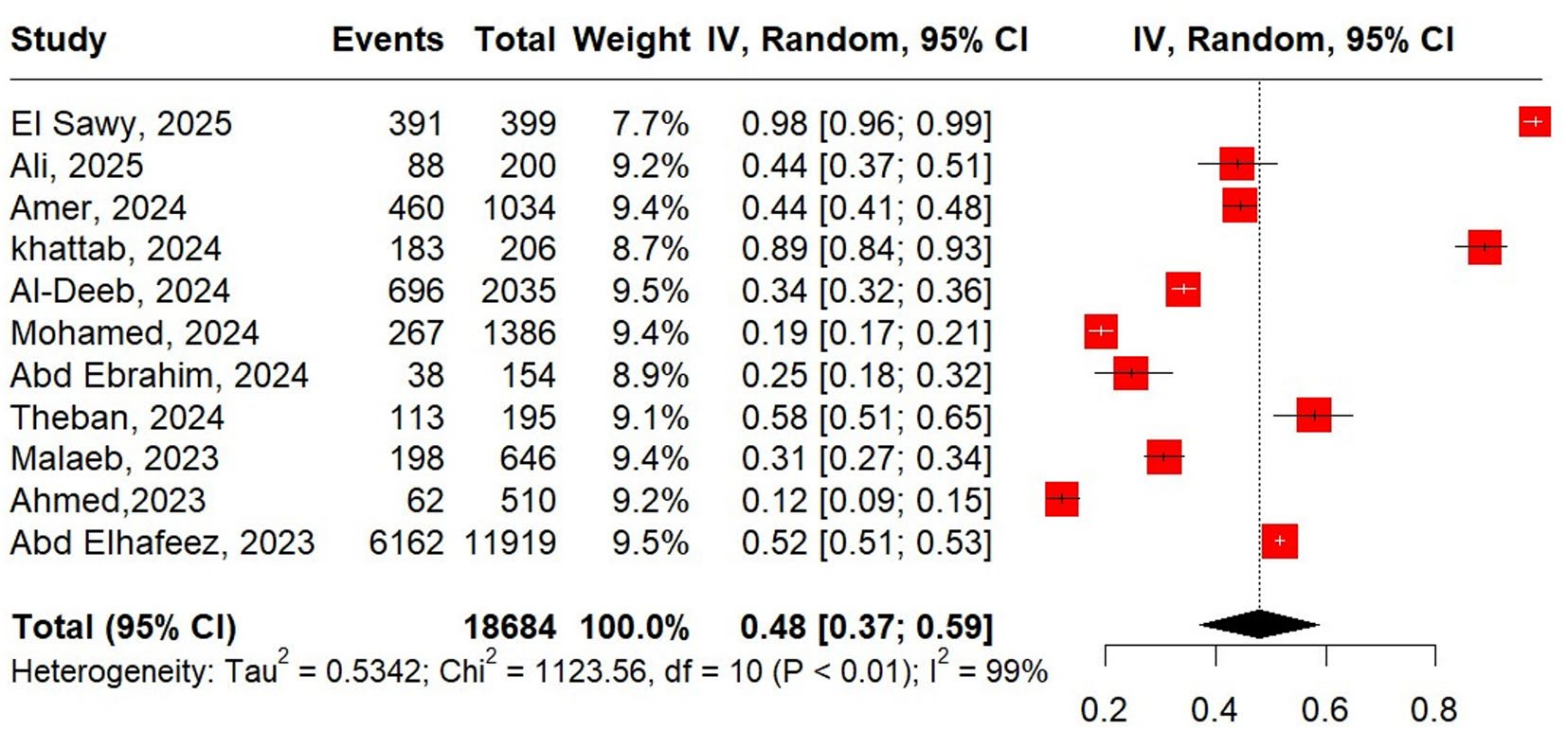
Forest plot of pooled positive attitude percentage among Arabic population.

Figure 4 presents a forest plot meta-analysis of attitudes toward Mpox in 18684 participants in Arab countries, showing a pooled positive attitude prevalence of 48% (95% CI: 37%−59%). The analysis demonstrates extreme heterogeneity (I^2^ = 99%) across the 11 included studies, with individual study results ranging from 12% (Ahmed, 2023) to 98% (ElSawy, 2025) positive attitudes.

Subgroup analyses identified population type and gender distribution as significant predictors of attitude variation. Healthcare workers showed markedly higher positive attitudes (79%, 95% CI: 56%−91%) compared to the general population (21%, 95% CI: 6%−54%) (p=0.020). Studies with predominantly female participants (>50% female) demonstrated significantly more positive attitudes (60%, 95% CI: 50%−69%) than male-dominated samples (p<0.001). Notably, while Egypt showed the highest attitude levels (75%, 95% CI: 49%−90%) despite having the lowest knowledge scores, this country-level difference was not statistically significant (p=0.150). Similarly, neither data collection year (p=0.080) nor mean participant age (p=0.770) showed significant associations with attitude levels, despite 2024 studies showing the highest attitude prevalence (70%, 95% CI: 47%−86%) and older populations (>30 years) demonstrating slightly more positive attitudes (51%, 95% CI: 36%−67%), Supplementary Table S6.6.- Supplementary Table S6.10.

## Discussion

This study uniquely combines a national Egyptian survey with a regional meta-analysis, providing the first comprehensive assessment of Mpox-related knowledge and attitudes among population in Arab countries. The dual-method design allows both national benchmarking and regional contextualization of results.

The Egyptian survey revealed that only 37.6% of HCWs demonstrated good knowledge, while 97.9% had a favorable attitude. This finding is consistent with the meta-analysis, which reported a pooled adequate knowledge of 35%, and a pooled positive attitude of 45% across Arab countries. This convergence indicates a persistent knowledge–attitude gap, where HCWs express willingness to act but lack the necessary understanding to do so effectively.

Our key finding of a significant gap in Mpox knowledge among HCWs aligns with the limited local studies reporting knowledge levels ranging from 7.3% to 55.3%^8,12^, as well as with the broader meta-analyses^14,15,19^. Similar findings have been reported in studies from various countries^7,9,10,20^. In contrast, a study from Nepal found that 53.8% of participants had good knowledge^21^, indicating that knowledge levels among HCWs in the Arab region remain comparatively lower. This regional deficit underscores the limited reach of educational initiatives and the delayed integration of Mpox training into continuing medical education programs. In Egypt, this may be partly due to the lack of formal Mpox education, reported by more than half of participants, and the country’s non-endemic status, which could reduce clinical vigilance and interest among medical staff^8^.

Interestingly, knowledge was not significantly associated with gender, age, or marital status, diverging from studies that reported higher knowledge among females^3,9^, or among males^22^. The strongest predictor of good knowledge was formal Mpox education (p < 0.001), emphasizing the impact of structured learning. Early career professionals (<1 year of experience) exhibited higher knowledge (59.3%) than those with >5 years (33.1%), possibly due to greater digital literacy and recent exposure to outbreak-related content^23,24^. This highlights a critical need for lifelong learning and periodic retraining for veteran practitioners^25^. Our meta-analysis revealed a notable rise in pooled knowledge from 25% in 2023 to 46% in 2024, suggesting increased awareness following the global outbreak and the scaling up of national response programs. Countries like Saudi Arabia, with greater research investment and exposure to mass gatherings (Hajj, Umrah), demonstrated higher knowledge, reflecting how epidemiological exposure stimulates preparedness^25^.

A striking finding was that Egyptian HCWs exhibited the most positive attitudes (75%) in the meta-analysis despite having the lowest knowledge scores. This optimism is also evident in our survey, where 97.9% held favorable views, aligning with Khattab et al.^8^, Sobaikhi et al.^18^, and opposite to Amer et al. and others^12,26,27^. Similar positivity (34.6%–71.9%) was reported in other meta-analyses^15,19^. The persistence of favorable attitudes may be linked to institutional trust, confidence in health authorities, and lessons learned from COVID-19^12^. However, attitudes without adequate knowledge may not translate into effective infection prevention practices.

Our findings also revealed gender disparities: male HCWs were more likely to hold negative attitudes, a trend supported by earlier work^28^, but contradicted by Tanashat et al.^29^. Such differences may stem from gendered variations in risk perception and health-seeking behavior, where females are generally more proactive and prevention-oriented^30^.

### Policy and educational implications

The findings underscore an urgent need for institutionalized educational reforms and policy-driven capacity building. Firstly, ministries of health should develop certified online training modules and standardized Mpox management protocols for HCWs, ensuring timely dissemination during outbreaks. Secondly, Medical and nursing schools should integrate emerging infectious disease modules into undergraduate curricula and adopt simulation-based outbreak preparedness training to enhance diagnostic confidence. Finally, public health authorities should conduct targeted awareness campaigns using social and traditional media to raise both HCW and community knowledge levels

### Strength and limitations

This study’s primary strength is the novel integration of a national survey with a meta-analysis, offering the first comprehensive regional assessment of Mpox knowledge and attitudes among HCWs in Arab countries. Furthermore, the inclusion of various HCWs categories and the independent nature of the data extraction for the meta-analysis ensure robustness. However, several limitations must be acknowledged. The cross-sectional, self-reported nature of the survey introduces information and social desirability biases. The online recruitment approach may have excluded HCWs without reliable internet access. In the meta-analysis, high heterogeneity and potential publication bias necessitate cautious interpretation. Furthermore, variations in assessment tools and populations mean the pooled estimates represent regional trends.

## Conclusion

This analysis shows consistent regional knowledge gaps despite favorable attitudes. The study highlights formal education and early career status as key determinants of knowledge, while gender differences shape attitudes. The main contribution of this research is its dual-method approach, which benchmarks Egypt’s performance within the broader Arab context, offering evidence-based insight for regional preparedness. To strengthen Mpox and outbreak response capacity, continuous professional development programs, curriculum modernization, and standardized training frameworks are urgently needed.

## Supplementary Information

Supplementary Information.

## Data Availability

The data used in this study will be available from the corresponding author upon reasonable request.

## Abbreviation

Mpox: Monkeypox
HCWs: Healthcare workers
MoHP: Ministry of health and population (Egypt)
KAP: Knowledge, attitudes, and practices
MSM: Men who have sex with men
WHO: World health organization
CHERRIES: Checklist for reporting results of internet E-surveys
MCQ: Multiple choice question
CAPMAS: Central agency for public mobilization and statistics
SD: Standard deviation
CI: Confidence interval
I^2^: I-squared (measure of heterogeneity in meta-analysis)
R: R statistical software
COVID-19: Coronavirus disease 2019
