# Supplementary material for: Assessment of Mpox knowledge and attitudes among health workers in Egypt and Arab countries based on a national survey and a meta-analysis
Source: Sci Rep. 2025 Dec 4;15:43249. doi: 10.1038/s41598-025-28446-z (PMC12680710; doi:10.1038/s41598-025-28446-z)
Supplement: Supplementary file 1 — Supplementary Information. [file 41598_2025_28446_MOESM1_ESM.docx]

***Phase 1 study:***


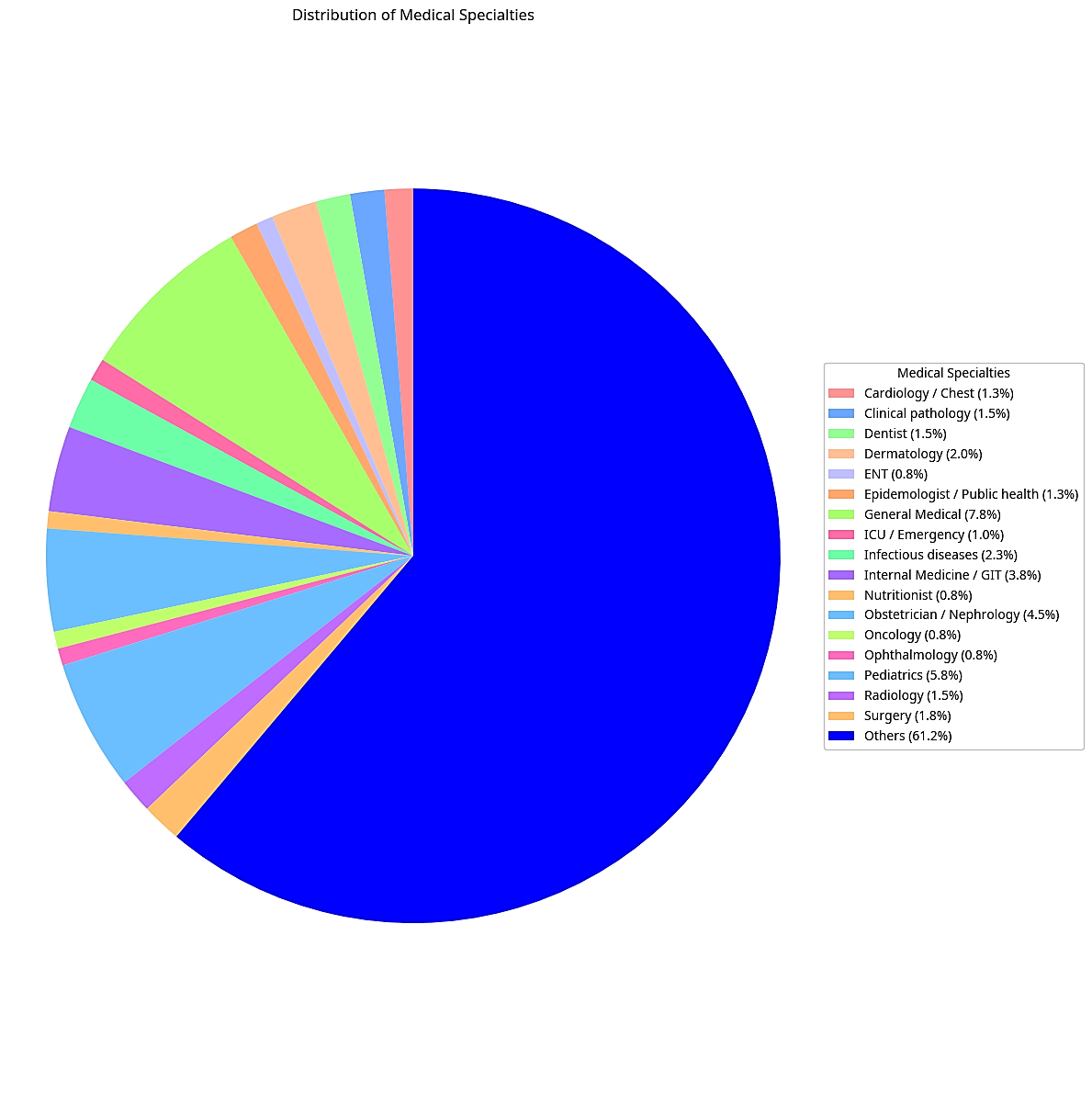


**Figure S1:** Pie chart of respondents' medical specialty distribution

**Table S2:** Knowledge assessment of M.Pox among healthcare workers by gender

| **Knowledge questions** | **Total** | **Female** | **Male** | **p-value** |
| --- | --- | --- | --- | --- |
| **Q1: Is M.Pox prevalent in middle eastern countries?** |  |  |  | **0.221** |
| Correct | 118 (29.6%) | 101 (30.9%) | 17 (23.6%) |  |
| Incorrect | 281 (70.4%) | 226 (69.1%) | 55 (76.4%) |  |
| **Q2: Is M.Pox prevalent in Western and Central Africa?** |  |  |  | **0.625** |
| Correct | 324 (81.2%) | 267 (81.7%) | 57 (79.2%) |  |
| Incorrect | 75 (18.8%) | 60 (18.3%) | 15 (20.8%) |  |
| **Q3: There are many human M.Pox cases in Egypt?** |  |  |  | **0.248** |
| Correct | 60 (15.0%) | 46 (14.1%) | 14 (19.4%) |  |
| Incorrect | 339 (85.0%) | 281 (85.9%) | 58 (80.6%) |  |
| **Q4: Is M.Pox a viral disease infection?** |  |  |  | **0.9** |
| Correct | 387 (97.0%) | 317 (96.9%) | 70 (97.2%) |  |
| Incorrect | 12 (3.0%) | 10 (3.1%) | 2 (2.8%) |  |
| **Q5: Is M.Pox a bacterial disease infection?** |  |  |  | **0.738** |
| Correct | 14 (3.5%) | 11 (3.4%) | 3 (4.2%) |  |
| Incorrect | 385 (96.5%) | 316 (96.6%) | 69 (95.8%) |  |
| **Q6: Is M.Pox easily transmitted human-to-human?** |  |  |  | **0.056** |
| Correct | 286 (71.7%) | 241 (73.7%) | 45 (62.5%) |  |
| Incorrect | 113 (28.3%) | 86 (26.3%) | 27 (37.5%) |  |
| **Q7: Could M.Pox be transmitted through bite of infected monkey?** |  |  |  | **0.473** |
| Correct | 253 (63.4%) | 210 (64.2%) | 43 (59.7%) |  |
| Incorrect | 146 (36.6%) | 117 (35.8%) | 29 (40.3%) |  |
| **Q8: Travelers from America and Europe are the primary source of imported cases of M.Pox?** |  |  |  | **0.219** |
| Correct | 209 (52.4%) | 176 (53.8%) | 33 (45.8%) |  |
| Incorrect | 190 (47.6%) | 151 (46.2%) | 39 (54.2%) |  |
| **Q9: Do M.Pox and smallpox have similar signs and symptoms?** |  |  |  | **0.546** |
| Correct | 282 (70.7%) | 229 (70.0%) | 53 (73.6%) |  |
| Incorrect | 117 (29.3%) | 98 (30.0%) | 19 (26.4%) |  |
| **Q10: A flu-like syndrome is one of the early signs or symptoms of human M.Pox?** |  |  |  | **0.503** |
| Correct | 333 (83.5%) | 271 (82.9%) | 62 (86.1%) |  |
| Incorrect | 66 (16.5%) | 56 (17.1%) | 10 (13.9%) |  |
| **Q11: Rashes on the skin are one of the signs or symptoms of human M.Pox?** |  |  |  | **0.376** |
| Correct | 370 (92.7%) | 305 (93.3%) | 65 (90.3%) |  |
| Incorrect | 29 (7.3%) | 22 (6.7%) | 7 (9.7%) |  |
| **Q12: Papules on the skin are one of the signs or symptoms of human M.Pox?** |  |  |  | **0.72** |
| Correct | 338 (84.7%) | 278 (85.0%) | 60 (83.3%) |  |
| Incorrect | 61 (15.3%) | 49 (15.0%) | 12 (16.7%) |  |
| **Q13: Vesicles on the skin are one of the signs or symptoms of human M.Pox?** |  |  |  | **0.654** |
| Correct | 334 (83.7%) | 275 (84.1%) | 59 (81.9%) |  |
| Incorrect | 65 (16.3%) | 52 (15.9%) | 13 (18.1%) |  |
| **Q14: Pustules on the skin are one of the signs or symptoms of human M.Pox?** |  |  |  | **0.675** |
| Correct | 297 (74.4%) | 242 (74.0%) | 55 (76.4%) |  |
| Incorrect | 102 (25.6%) | 85 (26.0%) | 17 (23.6%) |  |
| **Q15: Is diarrhea one of the signs or symptoms of human M.Pox?** |  |  |  | **0.055** |
| Correct | 181 (45.4%) | 141 (43.1%) | 40 (55.6%) |  |
| Incorrect | 218 (54.6%) | 186 (56.9%) | 32 (44.4%) |  |
| **Q16: Lymphadenopathy (swollen lymph nodes) is one clinical sign or symptom that could be used to differentiate between M.Pox and smallpox cases?** |  |  |  | **0.95** |
| Correct | 339 (85.0%) | 278 (85.0%) | 61 (84.7%) |  |
| Incorrect | 60 (15.0%) | 49 (15.0%) | 11 (15.3%) |  |
| **Q17: One management option for symptomatic M.Pox patients is to use paracetamol?** |  |  |  | **0.154** |
| Correct | 345 (86.5%) | 279 (85.3%) | 66 (91.7%) |  |
| Incorrect | 54 (13.5%) | 48 (14.7%) | 6 (8.3%) |  |
| **Q18: Are antivirals required in the management of human M.Pox patients?** |  |  |  | **0.122** |
| Correct | 305 (76.4%) | 255 (78.0%) | 50 (69.4%) |  |
| Incorrect | 94 (23.6%) | 72 (22.0%) | 22 (30.6%) |  |
| **Q19: Are antibiotics required in the management of human M.Pox patients?** |  |  |  | **0.023** |
| Correct | 78 (19.5%) | 57 (17.4%) | 21 (29.2%) |  |
| Incorrect | 321 (80.5%) | 270 (82.6%) | 51 (70.8%) |  |
| **Q20: People who got the chickenpox vaccine are immunized against M.Pox?** |  |  |  | **0.965** |
| Correct | 110 (27.6%) | 90 (27.5%) | 20 (27.8%) |  |
| Incorrect | 289 (72.4%) | 237 (72.5%) | 52 (72.2%) |  |
| **Q21: There is a specific vaccine for M.Pox?** |  |  |  | **0.869** |
| Correct | 114 (28.6%) | 94 (28.7%) | 20 (27.8%) |  |
| Incorrect | 285 (71.4%) | 233 (71.3%) | 52 (72.2%) |  |
| **Q22: There is a specific treatment for M.Pox?** |  |  |  | **0.896** |
| Correct | 91 (22.8%) | 75 (22.9%) | 16 (22.2%) |  |
| Incorrect | 308 (77.2%) | 252 (77.1%) | 56 (77.8%) |  |
| **Total knowledge** |  |  |  |  |
| Median (IQR) | 13.0 (11.0, 14.0) | 13.0 (11.5, 14.0) | 13.0 (11.0, 14.2) | **0.566** |
| Mean ± SD | 13.0 ± 2.6 | 13.0 ± 2.5 | 12.9 ± 3.0 | **0.897** |
| **Knowledge** |  |  |  | **0.774** |
| Good | 150 (37.6%) | 124 (37.9%) | 26 (36.1%) |  |
| Poor | 249 (62.4%) | 203 (62.1%) | 46 (63.9%) |  |

**Table S3:** Attitude assessment of M. Pox among healthcare workers by gender

| **Attitude questions** | **Total** | **Female** | **Male** | **p-value** |
| --- | --- | --- | --- | --- |
| **A1: I am confident that the world's population can control M.Pox worldwide** |  |  |  | **0.776** |
| Agree | 247 (61.9%) | 201 (61.5%) | 46 (63.9%) |  |
| Disagree | 18 (4.5%) | 14 (4.3%) | 4 (5.6%) |  |
| Neutral | 134 (33.6%) | 112 (34.3%) | 22 (30.6%) |  |
| **A2: I am confident that the Egyptian MoHP and local population can control the M.Pox locally** |  |  |  | **0.833** |
| Agree | 245 (61.4%) | 199 (60.9%) | 46 (63.9%) |  |
| Disagree | 37 (9.3%) | 30 (9.2%) | 7 (9.7%) |  |
| Neutral | 117 (29.3%) | 98 (30.0%) | 19 (26.4%) |  |
| **A3: I think that there are currently enough prevention and control measures for M.Pox** |  |  |  | **0.764** |
| Agree | 186 (46.6%) | 151 (46.2%) | 35 (48.6%) |  |
| Disagree | 71 (17.8%) | 57 (17.4%) | 14 (19.4%) |  |
| Neutral | 142 (35.6%) | 119 (36.4%) | 23 (31.9%) |  |
| **A4: I think M.Pox can be transmitted to Egypt** |  |  |  | **0.717** |
| Agree | 201 (50.4%) | 162 (49.5%) | 39 (54.2%) |  |
| Disagree | 60 (15.0%) | 51 (15.6%) | 9 (12.5%) |  |
| Neutral | 138 (34.6%) | 114 (34.9%) | 24 (33.3%) |  |
| **A5: I think that mass media coverage of M.Pox may influence worldwide prevention** |  |  |  | **0.011*** |
| Agree | 270 (67.7%) | 211 (64.5%) | 59 (81.9%) |  |
| Disagree | 25 (6.3%) | 24 (7.3%) | 1 (1.4%) |  |
| Neutral | 104 (26.1%) | 92 (28.1%) | 12 (16.7%) |  |
| **A6: I am interested in learning more about M.Pox** |  |  |  | **0.413** |
| Agree | 317 (79.4%) | 261 (79.8%) | 56 (77.8%) |  |
| Disagree | 20 (5.0%) | 18 (5.5%) | 2 (2.8%) |  |
| Neutral | 62 (15.5%) | 48 (14.7%) | 14 (19.4%) |  |
| **A7: I have bad feelings toward the M.Pox virus that it might become a worldwide pandemic** |  |  |  | **0.465** |
| Agree | 138 (34.6%) | 116 (35.5%) | 22 (30.6%) |  |
| Disagree | 125 (31.3%) | 104 (31.8%) | 21 (29.2%) |  |
| Neutral | 136 (34.1%) | 107 (32.7%) | 29 (40.3%) |  |
| **A8: I think that M.Pox can add a new burden on the healthcare system of the affected countries** |  |  |  | **0.045*** |
| Agree | 226 (56.6%) | 193 (59.0%) | 33 (45.8%) |  |
| Disagree | 45 (11.3%) | 38 (11.6%) | 7 (9.7%) |  |
| Neutral | 128 (32.1%) | 96 (29.4%) | 32 (44.4%) |  |
| **A9: I am interested to learn more about the epidemiology of the emerging diseases** |  |  |  | **0.301** |
| Agree | 300 (75.2%) | 251 (76.8%) | 49 (68.1%) |  |
| Disagree | 22 (5.5%) | 17 (5.2%) | 5 (6.9%) |  |
| Neutral | 77 (19.3%) | 59 (18.0%) | 18 (25.0%) |  |
| **A10: I am interested in learning more about Travel Medicine** |  |  |  | **0.891** |
| Agree | 298 (74.7%) | 245 (74.9%) | 53 (73.6%) |  |
| Disagree | 25 (6.3%) | 21 (6.4%) | 4 (5.6%) |  |
| Neutral | 76 (19.0%) | 61 (18.7%) | 15 (20.8%) |  |
| **A11: I think that it is dangerous to travel to the country's epidemic M.Pox** |  |  |  | **0.027*** |
| Agree | 267 (66.9%) | 226 (69.1%) | 41 (56.9%) |  |
| Disagree | 36 (9.0%) | 24 (7.3%) | 12 (16.7%) |  |
| Neutral | 96 (24.1%) | 77 (23.5%) | 19 (26.4%) |  |
| **Attitude** |  |  |  | **0.643** |
| Positive | 391 (97.9%) | 320 (97.9%) | 71 (98.6%) |  |

***Phase 2 study***

**Table S4:** Search strategy and selection process for studies on knowledge and attitudes toward M.Pox (Mpox) in Arab countries (2022–2025)

| **Database** | **Search Strategy / Keywords** | **Date of Search** | **Initial Hits** | **After Filters (English + Humans)** | **Manual Additions** | **Irrelevant / Excluded** | **Final Included Studies** |
| --- | --- | --- | --- | --- | --- | --- | --- |
| **PubMed** | **("M.Pox" OR "Mpox" OR "M.Pox virus") AND ("knowledge" OR "awareness" OR "attitude" OR "perception" OR "practice" OR "KAP") AND ("Saudi Arabia" OR "Egypt" OR "Jordan" OR "Kuwait" OR "United Arab Emirates" OR "UAE" OR "Qatar" OR "Bahrain" OR "Oman" OR "Lebanon" OR "Syria" OR "Iraq" OR "Palestine" OR "Sudan" OR "Yemen" OR "Libya" OR "Tunisia" OR "Algeria" OR "Morocco" OR "Comoros" OR "Mauritania" OR "Djibouti" OR "Somalia")** | **May 3, 2025 (from 2022 till May 2025)** | **135** | **65** | **5** | **40** | **30** |

**Table S5:** Characteristics of the included studies on knowledge and attitudes toward M.Pox

|  | **Author** | **Year** | **Study period** | **Country** | **Poulation** | **Male %** | **Mean Age** | **Good knowledge %** | **Good attitude%** |
| --- | --- | --- | --- | --- | --- | --- | --- | --- | --- |
| 1 | **El Sawy** | **2025** | **2024** | **Egypt** | **HCWs** | **18%** | **35.6** | **37.6** | **97.9** |
| 2 | **Mohamed**(1) | **2025** | **December 2022 to February 2023)** | **Saudia Arabia** | **HCWs and medical students** | **48.50%** | **35** | **3.9 (score > 17)** | **NA** |
| 3 | **Ali**(2) | **2025** | **April 2024 to June 2024.** | **Saudia Arabia** | **Healthcare students** | **16.50%** | **21** | **32.0** | **44.0** |
| 4 | **Alshammari**(3) | **2025** | **1st September 2024 till 31st January 2025** | **Saudia Arabia** | **Healthcare students** | **31.10%** | **21** | **71.9** | **NA** |
| 5 | **Amer**(4) | **2024** | **October and December 2022** | **Egypt** | **HCWs and medical students** | **40.40%** | **30** | **55.3** | **44.5** |
| 6 | **khattab**(5) | **2024** | **October 2022 and November 2022** | **Egypt** | **HCWs** | **17.50%** | **31 ± 6.7** | **7.3** | **89.3** |
| 7 | **Al-Deeb**(6) | **2024** | **October 25 and November 22, 2024** | **Syria** | **General population** | **36.20%** | **30** | **41.1** | **34.2** |
| 8 | **Dabou**(7) | **2024** | **august 2023 to November 2023** | **United Arab Emirates** | **Healthcare students** | **24.10%** | **21.2 ± 2.8** | **22.1** | **NA** |
| 9 | **Mohamed**(8) | **2024** | **October 2022 to February 2023.** | **Several countries** | **Healthcare students** | **65.00%** | **23.7 ± 5.7** | **21.8** | **19.3** |
| 10 | **Abd Ebrahim**(9) | **2024** | **March till the end of May 2023** | **Egypt** | **Healthcare students** | **61.69%** | **19.4** | **20.0** | **25.0** |
| 11 | **Theban**(10) | **2024** | **November 1 and December 31, 2022.** | **Saudia Arabia** | **HCWs** | **43.60%** | **35** | **16.9** | **57.9** |
| 12 | **Sobaikhi**(11) | **2023** | **4 November 2022 - 8 December 2022** | **Saudia Arabia** | **HCWs** | **61.8** | **30.93 ± 8.25** | **44.1** | **NA** |
| 13 | **Swed1**(12) | **2023** | **June 6 to June 25, 2022** | **Several countries** | **HCWs** | **41.8** | **30** | **36.0** | **NA** |
| 14 | **Malaeb**(13) | **2023** | **september-Dercember 2022** | **Lebanon** | **HCWs** | **46.7** | **32.64 ± 10.08** | **33.7** | **30.7** |
| 15 | **Swed2**(14) | **2023** | **May 2 & september 8, 2022** | **Syria** | **HCWs and medical students** | **38.3** | **30** | **23.0** | **NA** |
| 16 | **Youssef**(15) | **2023** | **over the first 2Â weeks of August 2022** | **Lebanon** | **General population** | **29.13** | **30** | **33.0** | **NA** |
| 17 | **Elkhwesky**(16) | **2023** | **September , 2022** | **Egypt** | **General population** | **80.10%** | **25-45** | **18.1** | **NA** |
| 18 | **Swed3**(17) | **2023** | **18 August and 7 September 2022,** | **Several countries** | **General population** | **40.30%** | **32** | **45.5** | **NA** |
| 19 | **Ahmed**(18) | **2023** | **27-30 July 2022** | **Yemen / Iraq** | **General population** | **54.30%** | **31.1 ± 8.5** | **40.4** | **12.2** |
| 20 | **Abd Elhafeez**(19) | **2023** | **September 1 to December 15, 2022.** | **Several countries** | **Healthcare students** | **46%** | **21.7 ± 2.2** | **55.3** | **51.7** |
| 21 | **Zeidan**(20) | **2023** | **Jun-22** | **United Arab Emirates** | **General population** | **29%** | **34** | **56.2** | **NA** |
| 22 | **Halboup**(21) | **2023** | **December 2022 to March 2023** | **Yemen / Iraq** | **General population** | **60.50%** | **23(IQR,7)** | **32.9** | **NA** |
| 23 | **Gebreal**(22) | **2023** | **August-October, 2022** | **Several countries** | **Healthcare students** | **37%** | **21** | **35.5** | **NA** |
| 24 | **Alshahrani 1**(23) | **2022** | **26 March 2022 to 27 May 2022.** | **Saudia Arabia** | **HCWs** | **56.8** | **35** | **55.0** | **NA** |
| 25 | **Ajamaan**(24) | **2022** | **May 27-10 June 2022** | **Saudia Arabia** | **HCWs** | **37.3** | **37.1 ± 9.69** | **23.2** | **NA** |
| 26 | **Alshahrani 2** (25) | **2022** | **May 25-July 15, 2022** | **Saudia Arabia** | **General population** | **41.3** | **30 ± 10.07** | **48.0** | **NA** |
| 27 | **Alshahrani 3** (26) | **2022** | **May 24- July 20, 2022** | **Saudia Arabia** | **Healthcare students** | **41.70%** | **21** | **28.0** | **NA** |
| 28 | **Temsah**(27) | **2022** | **May 27 and June 5, 2022** | **Saudia Arabia** | **General population** | **42%** | **23** | **56.0** | **NA** |
| 29 | **Naik**(28) | **2025** | **August and November 2022** | **saudi arabia** | **HCWs** | **34%** | **18-29** | **36.1** | **NA** |
| 30 | **Peer-Zada**(29) | **2025** | **December 2022 to February 2023** | **saudi arabia** | **medical students** | **39%** | **18-27** | **16.0** | **NA** |

**S6: Subgroup analysis:**

S 6.1.: knowledge subgrouping by year of collecting data


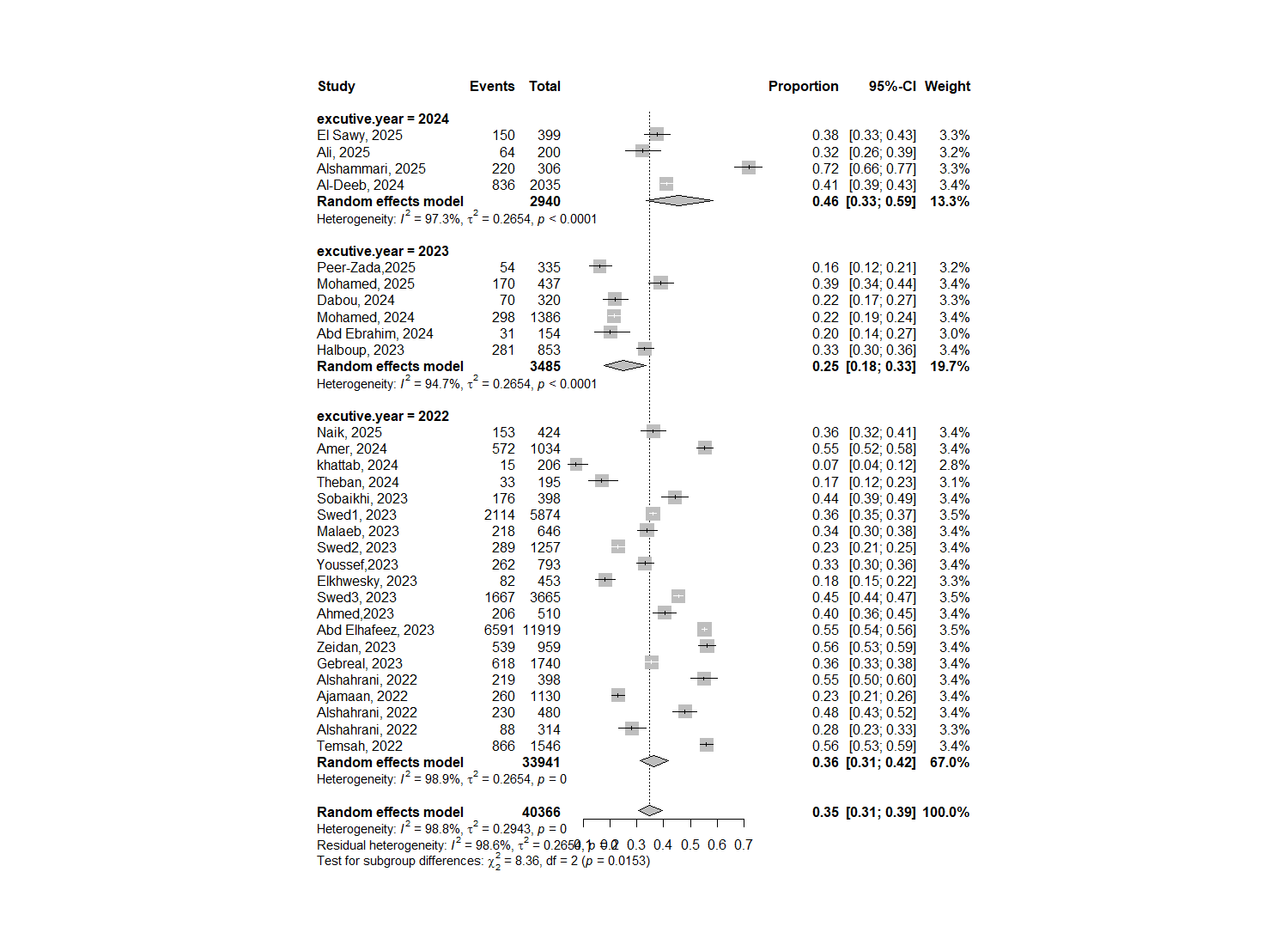


S 6.2.: knowledge subgrouping by country
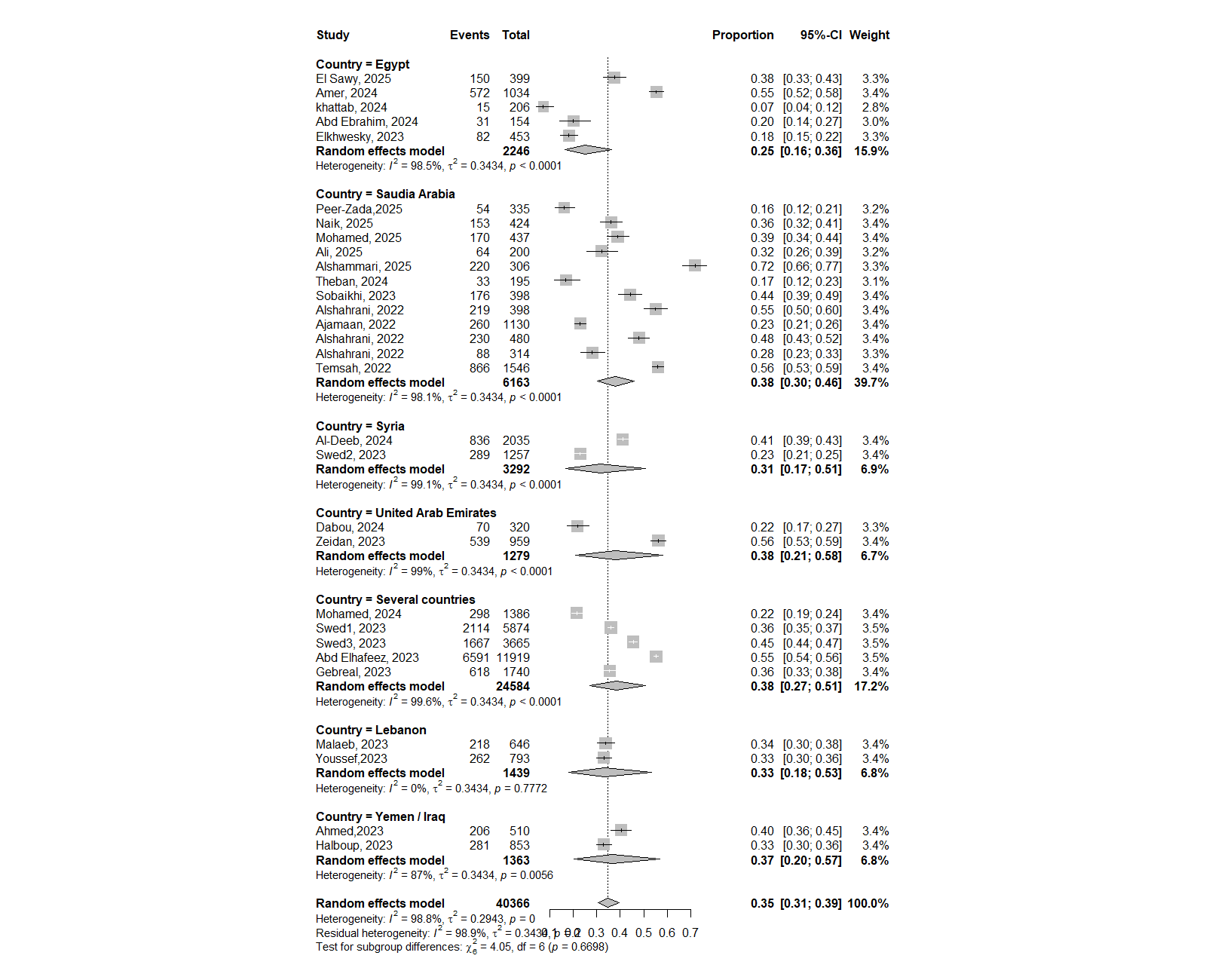


S 6.3.: knowledge subgrouping by population


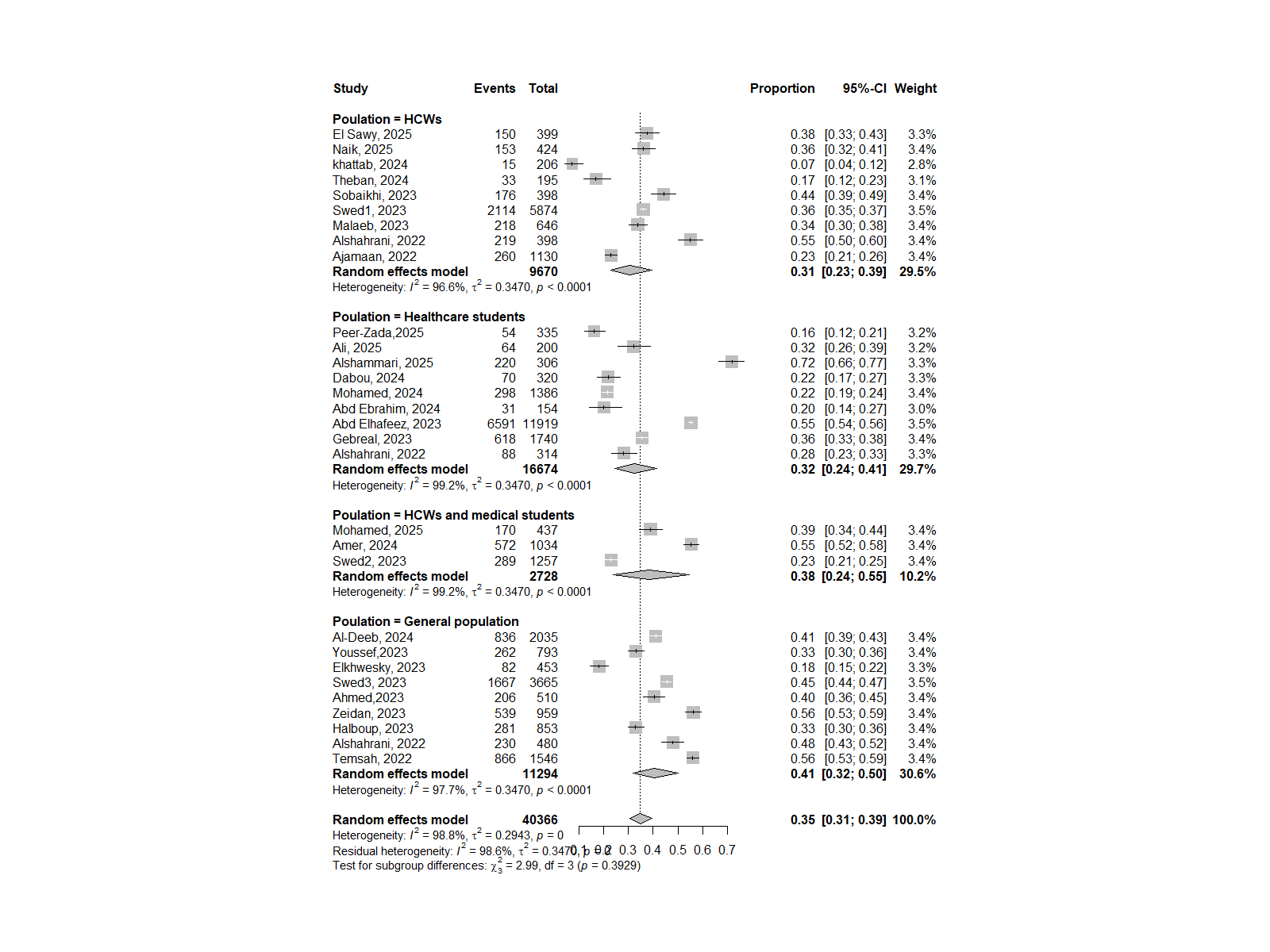


S 6.4.: knowledge subgrouping by gender (female percentage of total respondent)
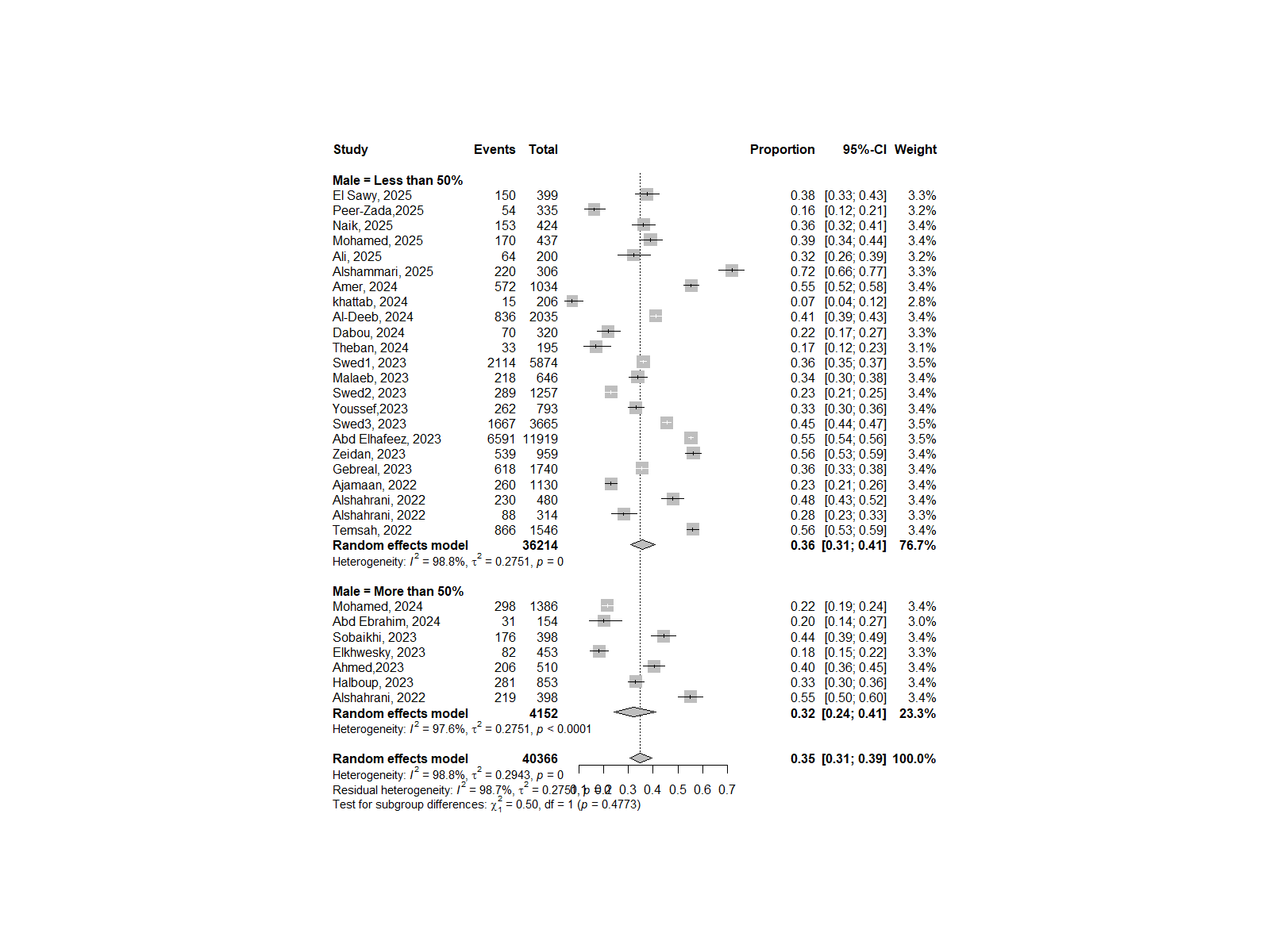


S 6.5.: knowledge subgrouping by mean age of respondents


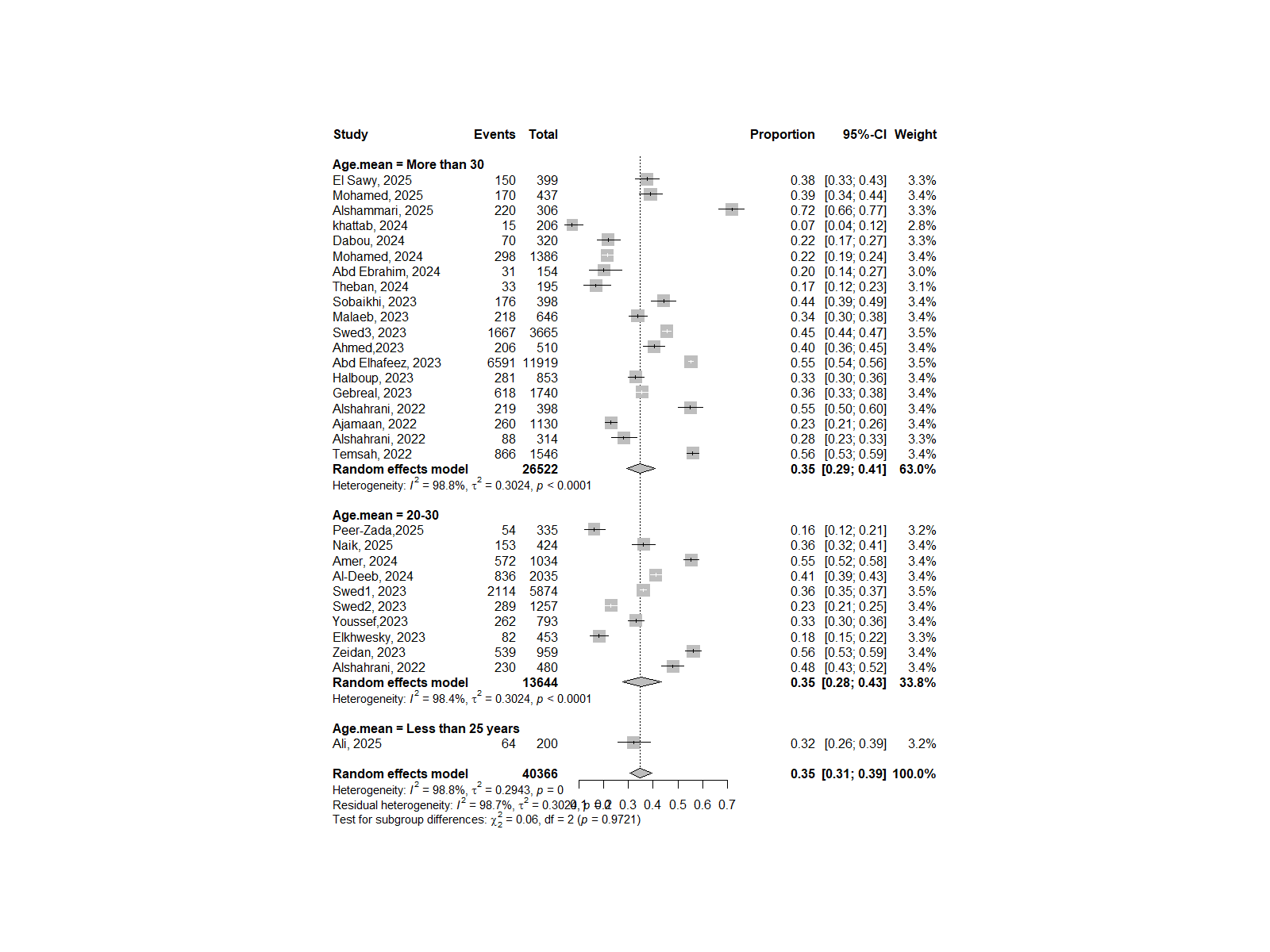


S 6.6.: Attitude subgrouping by year of data collection


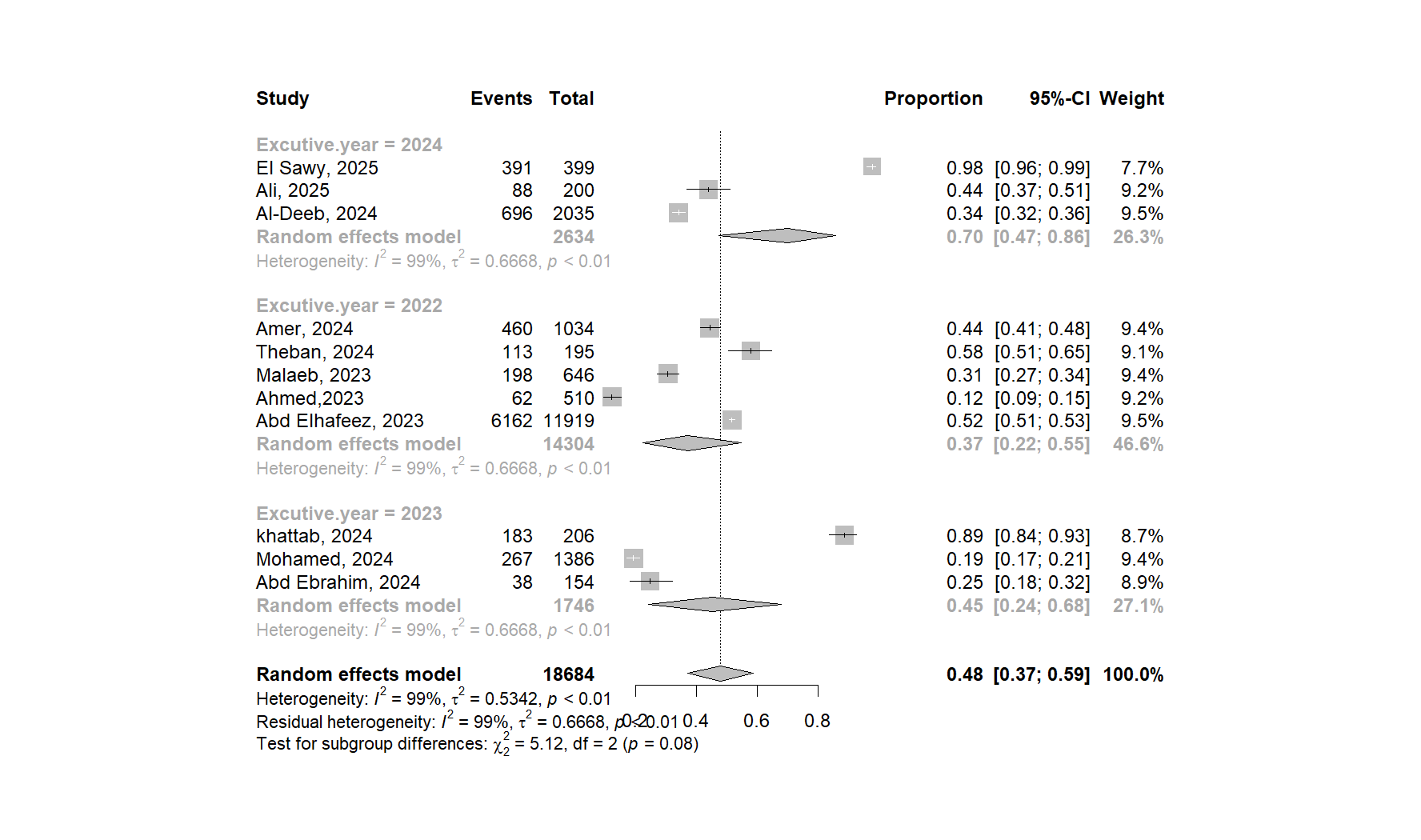


S 6.7.: Attitude subgrouping by country


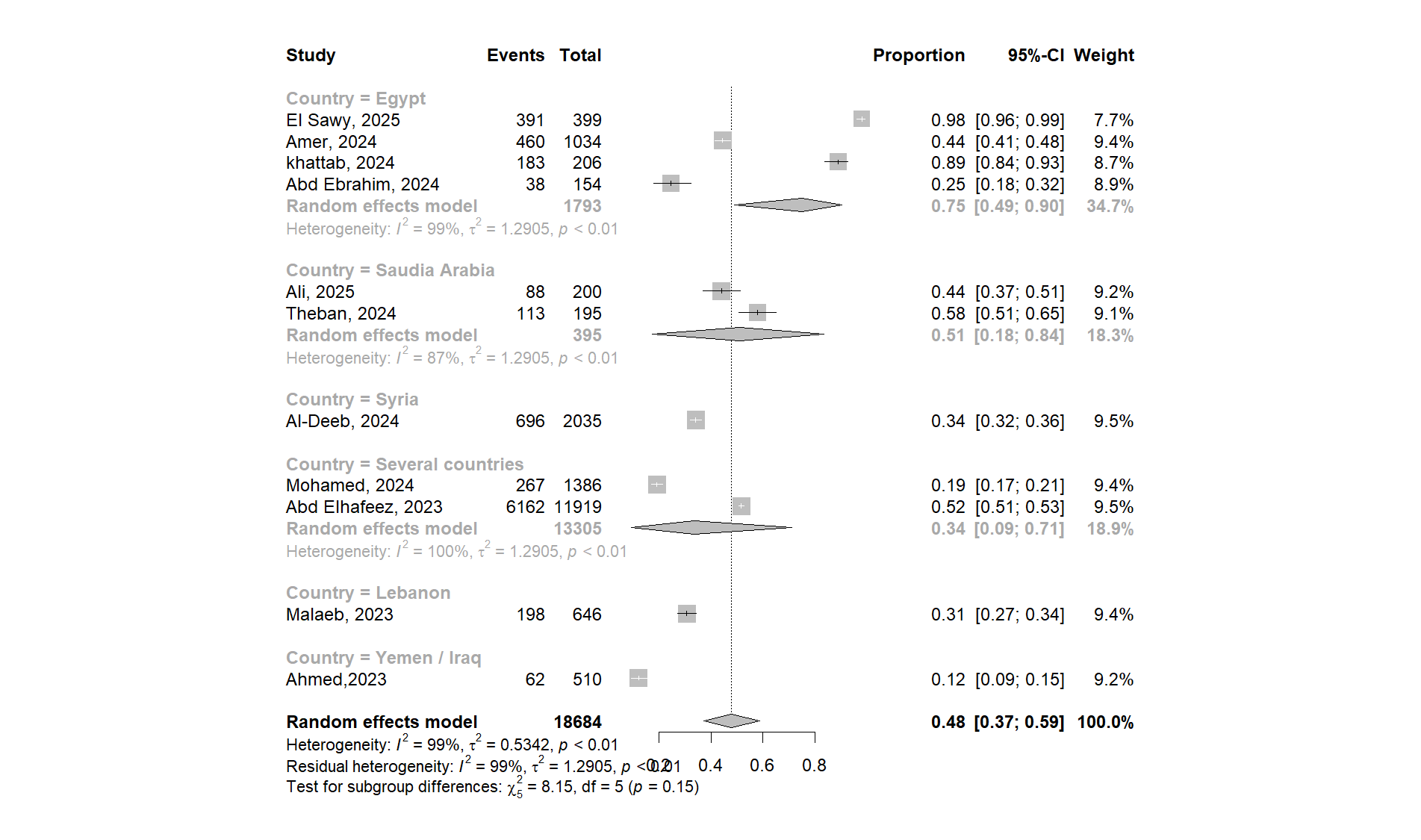


S 6.8.: Attitude subgrouping by population


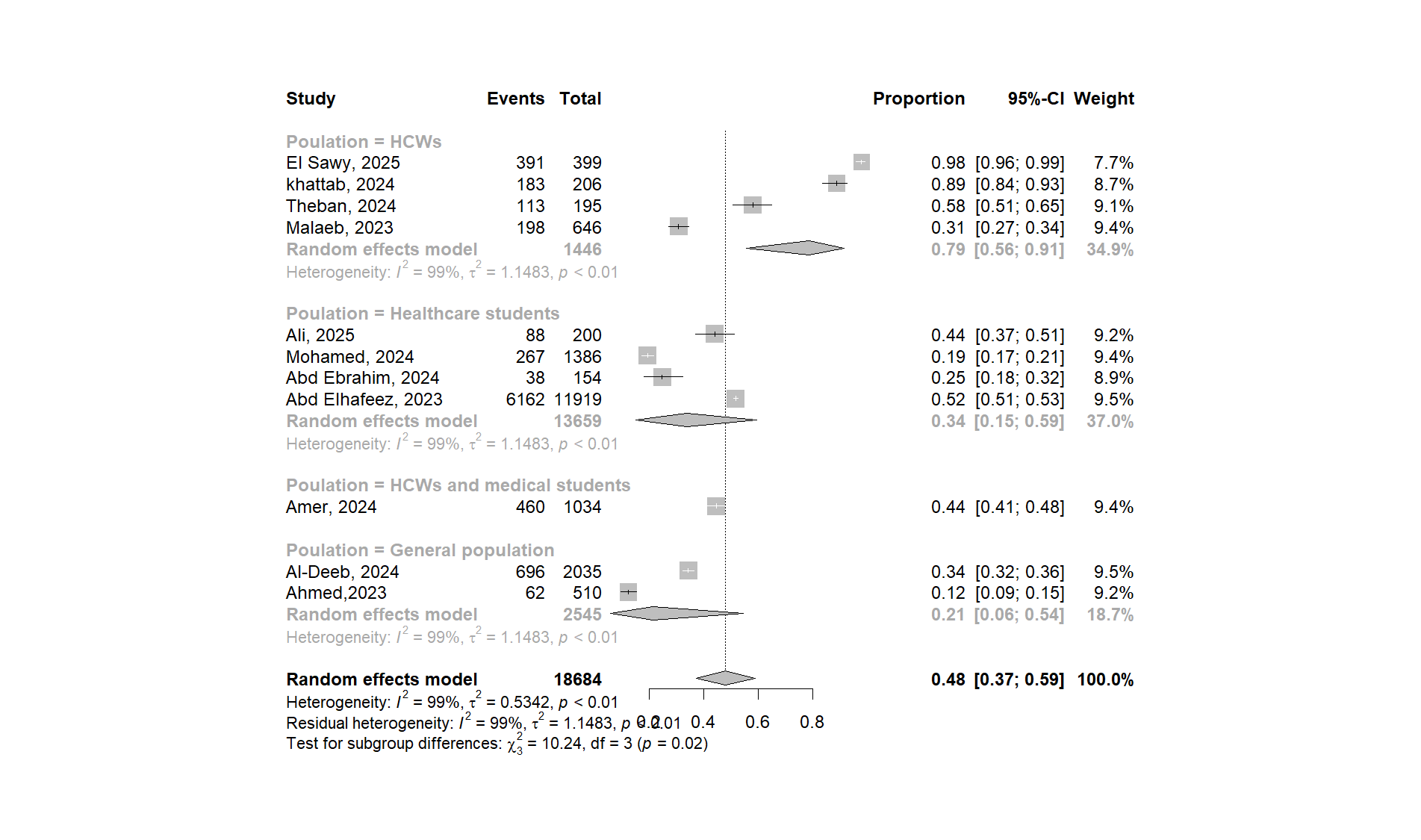


S 6.9.: Attitude subgrouping by female percentage per total respondents
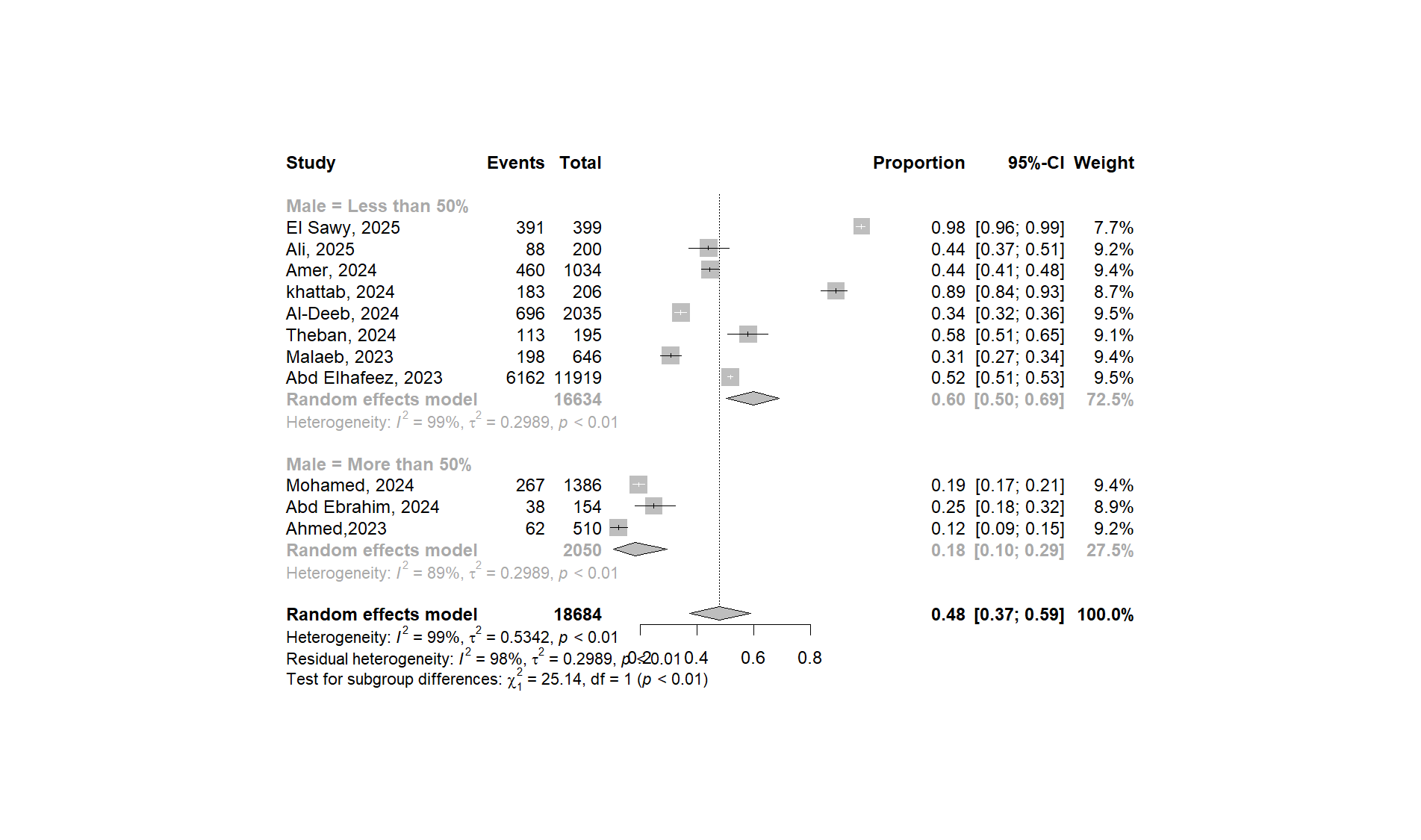


S 6.10.: Attitude subgrouping by mean age


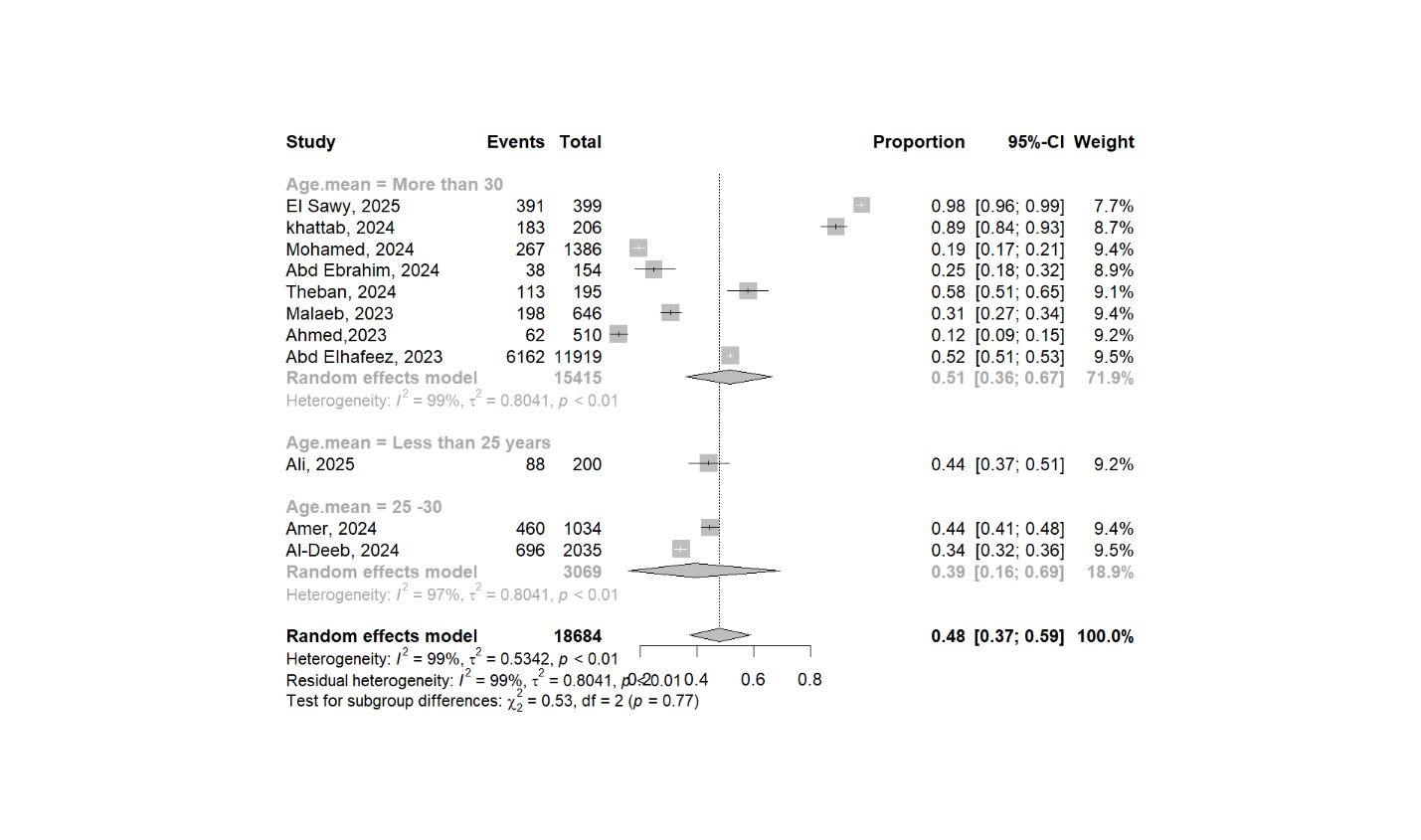


**References:**

1. Mohamed MS, Idriss MT, Alotaibi NH, Khan YH, Mallhi TH. Appraisal of healthcare students and professionals’ knowledge and intention to educate the public regarding M.Pox (Mpox). PeerJ. 2025;13(4).

2. Ali EM, Khardali A, Alam N, Meraya AM, Thaibah HA, Hassan DA, et al. Knowledge and attitude of human Mpox viral infection among pharmacy students in Jazan University: a web based cross-sectional study. Front Public Heal [Internet]. 2025 Apr 25;13. Available from: https://www.frontiersin.org/articles/10.3389/fpubh.2025.1521923/full

3. Saqr I, Alshammari F, Saleh S, Alanazi S, Zafar M, Sayedalameen AAM, et al. Monkey pox preparedness : evaluating awareness and knowledge among healthcare students , Hail Saudi Arabia. 2025;9(March):733–9.

4. Amer FA, Nofal HA, Gebriel MG, Bedawy AM, Allam AA, Khalil HES, et al. Grasping knowledge, attitude, and perception towards M.Pox among healthcare workers and medical students: an Egyptian cross-sectional study. Front Cell Infect Microbiol. 2024;14(February):1–10.

5. Khattab sally, Nemr NN, Kishk RM, Mohamed HM, Nemr NN, El-Ghazaly AA, et al. Knowledge and attitude towards Mpox among healthcare workers: an intervention study. Microbes Infect Dis. 2024 Jul 28;0(0):0–0.

6. Al-Deeb R, Wali A, Mansour N, Shamme N, Almansour S, Ataya J, et al. M.Pox awareness and public health challenges in conflict affected syria 2024. Sci Rep [Internet]. 2025 Dec 1 [cited 2025 Jul 15];15(1). Available from: https://pubmed.ncbi.nlm.nih.gov/39930074/

7. Abdelaziz Rashad Dabou E, Magdi Ibrahim F, Ekama Ilesanmi R. Knowledge, Awareness, and perceived anxiety of human M.Pox among university students in Ras al Khaimah, United Arab of Emirates. Int J Africa Nurs Sci [Internet]. 2024;20(June):100743. Available from: https://doi.org/10.1016/j.ijans.2024.100743

8. Mohamed MG, Islam MR, Ahmed SK, Khalaf SA, Abdelall HA, Mahmood KA, et al. Assessment of knowledge, attitude, anxiety level and perceived mental healthcare needs toward mpox infection among nursing students: A multi-center cross-sectional study. Glob Transitions [Internet]. 2024;6:203–11. Available from: https://doi.org/10.1016/j.glt.2024.10.001

9. Abd Elrahim ZM. Nursing student perception of monkey pox virus at the technical health institute of imbaba. Helwan Int J Nurs Res Pract. 2024;3(6):179–93.

10. Chowdhury S, Chakraborty P pratim. Universal health coverage ‑ There is more to it than meets the eye. J Fam Med Prim Care [Internet]. 2017;6(2):169–70. Available from: http://www.jfmpc.com/article.asp?issn=2249-4863;year=2017;volume=6;issue=1;spage=169;epage=170;aulast=Faizi

11. Sobaikhi NH, Alshahrani NZ, Hazazi RS, Al-Musawa HI, Jarram RE, Alabah AE, et al. Health Workers’ Knowledge and Attitude towards M.Pox in Southwestern Saudi Arabia: A Cross-Sectional Study. Diseases. 2023 Jun 1;11(2).

12. Swed S, Bohsas H, Patwary MM, Alibrahim H, Rakab A, Nashwan AJ, et al. Knowledge of mpox and its determinants among the healthcare personnel in Arabic regions: A multi-country cross-sectional study. New Microbes New Infect. 2023 Sep 1;54.

13. Malaeb D, Sallam M, Salim NA, Dabbous M, Younes S, Nasrallah Y, et al. Knowledge, Attitude and Conspiracy Beliefs of Healthcare Workers in Lebanon towards M.Pox. Trop Med Infect Dis. 2023 Feb 1;8(2).

14. Swed S, Alibrahim H, Bohsas H, Aljabali A, Almoshantaf MB, Sawaf B, et al. M.Pox in Syria: Highlighting an awareness issue. IJID Reg. 2023 Jun 1;7:271–6.

15. Youssef D, Abboud E, Kawtharani M, Zheim Z, Abou Arrage N, Youssef J. When a neglected tropical zoonotic disease emerges in non-endemic countries: need to proactively fill the unveiled knowledge gaps towards human M.Pox among the Lebanese population. J Pharm Policy Pract [Internet]. 2023;16(1):1–20. Available from: https://doi.org/10.1186/s40545-023-00544-1

16. Elkhwesky Z, Derhab N, Elkhwesky FFY, Abuelhassan AE, Hassan H. Hotel employees’ knowledge of M.Pox’s source, symptoms, transmission, prevention, and treatment in Egypt. Travel Med Infect Dis [Internet]. 2023;53(April):102574. Available from: https://doi.org/10.1016/j.tmaid.2023.102574

17. Swed S, Bohsas H, Alibrahim H, Rakab A, Hafez W, Sawaf B, et al. M.Pox Post-COVID-19: Knowledge, Worrying, and Vaccine Adoption in the Arabic General Population. Vaccines. 2023 Apr 1;11(4).

18. Ahmed SK, Abdulqadir SO, Omar RM, Abdullah AJ, Rahman HA, Hussein SH, et al. Knowledge, Attitude and Worry in the Kurdistan Region of Iraq during the Mpox (M.Pox) Outbreak in 2022: An Online Cross-Sectional Study. Vaccines. 2023 Mar 1;11(3).

19. Abd ElHafeez S, Gebreal A, Khalil MA, Youssef N, Sallam M, Elshabrawy A, et al. Assessing disparities in medical students’ knowledge and attitude about M.Pox: a cross-sectional study of 27 countries across three continents. Front Public Heal. 2023;11(July):1–14.

20. Zeidan RK, Shukla A, Hussein A, AlZubaidi H, Temsah MH, AlHajjaj MS, et al. Assessment of Knowledge, Perceptions, and Attitudes During the Global Mpox Outbreak in June 2022: A Cross-Sectional Study From the United Arab Emirates. Int J Public Health. 2023;68(November):1–13.

21. Halboup AM, Alzoubi KH, Abu-Farha RK, Harun SN, Al-Mohamadi A, Battah M, et al. Perceptions and Knowledge of Public Towards Emerging Human M.Pox in Yemen: A Cross-Sectional Study [Response to Letter]. J Multidiscip Healthc. 2024;17(November):55–6.

22. Gebreal A, ElHafeez SA, Abdelmoneim SA, Ghazy RM. Knowledge of M.Pox among medical students in the Eastern Mediterranean Region. Popul Med [Internet]. 2023 Apr 27 [cited 2025 Jul 15];5(Supplement). Available from: https://www.populationmedicine.eu/Knowledge-of-M.Pox-among-medical-students-in-the-Eastern-Mediterranean-Region,165096,0,2.html

23. Alshahrani NZ, Algethami MR, Alarifi AM, Alzahrani F, Alshehri EA, Alshehri AM, et al. Knowledge and Attitude Regarding M.Pox Virus among Physicians in Saudi Arabia: A Cross-Sectional Study. Vaccines. 2022 Dec 1;10(12).

24. Ajman F, Alenezi S, Alhasan K, Saddik B, Alhaboob A, Altawil ES, et al. Healthcare Workers’ Worries and M.Pox Vaccine Advocacy during the First Month of the WHO M.Pox Alert: Cross-Sectional Survey in Saudi Arabia. Vaccines. 2022;10(9).

25. Alshahrani NZ, Alzahrani F, Alarifi AM, Algethami MR, Alhumam MN, Ayied HAM, et al. Assessment of Knowledge of M.Pox Viral Infection among the General Population in Saudi Arabia. Pathogens. 2022 Aug 1;11(8).

26. Alshahrani NZ, Mitra S, Alkuwaiti AA, Alhumam MN, Altmimi SMB, Alamri MHM, et al. Medical Students’ Perception Regarding the Re-emerging M.Pox Virus: An Institution-Based Cross-Sectional Study From Saudi Arabia. Cureus. 2022 Aug 16;

27. Temsah MH, Aljamaan F, Alenezi S, Alhasan K, Saddik B, Al-Barag A, et al. M.Pox caused less worry than COVID-19 among the general population during the first month of the WHO M.Pox alert: Experience from Saudi Arabia. Travel Med Infect Dis [Internet]. 2022;49(August):102426. Available from: https://doi.org/10.1016/j.tmaid.2022.102426

28. Naik S, Vellappally S, Al Kheraif AA, Alsarani MM, Basavarajappa S, Alhassoun RK, et al. M.Pox Virus Outbreak 2022: Assessment of Knowledge Among Healthcare Workers in Two Countries—India and Saudi Arabia. Int J Clin Pract. 2025;2025(1).

29. Peer-Zada F, Mahmoud H, Rahman H, Abdulmowla OM, Mohamed S, Raziq T, et al. Knowledge, attitude, and practice of medical students toward Mpox in Saudi Arabia: a cross-sectional study. Ann Med Surg [Internet]. 2025 Mar;87(3):1200–8. Available from: https://journals.lww.com/10.1097/MS9.0000000000002935
